# Multielectron transportation of polyoxometalate-grafted metalloporphyrin coordination frameworks for selective CO_2_-to-CH_4_ photoconversion

**DOI:** 10.1093/nsr/nwz096

**Published:** 2019-07-16

**Authors:** Qing Huang, Jiang Liu, Liang Feng, Qi Wang, Wei Guan, Long-Zhang Dong, Lei Zhang, Li-Kai Yan, Ya-Qian Lan, Hong-Cai Zhou

**Affiliations:** 1 Jiangsu Collaborative Innovation Centre of Biomedical Functional Materials, Jiangsu Key Laboratory of New Power Batteries, School of Chemistry and Materials Science, Nanjing Normal University, Nanjing 210023, China; 2 Department of Chemistry, Northeast Normal University, Changchun 130024, China; 3 Department of Chemistry, Texas A&M University, College Station, TX 77843-3255, USA

**Keywords:** reductive polyoxometalate, coordination frameworks, CO_2_ photoreduction, high selectivity, methane

## Abstract

Photocatalytic CO_2_ reduction into energy carriers is of utmost importance due to the rising concentrations of CO_2_ and the depleting energy resource. However, the highly selective generation of desirable hydrocarbon fuel, such as methane (CH_4_), from CO_2_ remains extremely challenging. Herein, we present two stable polyoxometalate-grafted metalloporphyrin coordination frameworks (POMCFs), which are constructed with reductive Zn-ϵ-Keggin clusters and photosensitive tetrakis(4-carboxylphenyl)porphyrin (H_2_TCPP) linkers, exhibiting high selectivity (>96%) for CH_4_ formation in a photocatalytic CO_2_-reduction system. To our knowledge, the high CH_4_ selectivity of POMCFs has surpassed all of the reported coordination-framework-based heterogeneous photocatalysts for CO_2_-to-CH_4_ conversion. Significantly, the introduction of a Zn-ϵ-keggin cluster with strong reducing ability is the important origin for POMCFs to obtain high photocatalytic selectivity for CH_4_ formation, considering that eight Mo^V^ atoms can theoretically donate eight electrons to fulfill the multielectron reduction process of CO_2_-to-CH_4_ transformation.

## INTRODUCTION

Excessive CO_2_ discharge derived from the continuous burning of fossil fuels has caused global warming and environmental issues [1,2]. Artificial conversion of excess CO_2_ into a serviceable energy product is an important pathway to achieve sustainable development [3–6]. Solar-driven photocatalytic reduction of CO_2_ to carbon-neutral fuels (CO, CH_4_) and/or value-added chemicals (HCOOH, CH_3_OH) affords a feasible strategy for the aforesaid conversion [7–11]. The implementation of this reaction can mitigate the greenhouse effect and energy crisis simultaneously. However, the structural activation process of the CO_2_ molecule is particularly difficult because of its intrinsically chemical inertness and high C=O bond-cleavage enthalpy [12]. In order to circumvent the highly negative equilibrium potential versus normal hydrogen electrode (NHE) for thermodynamically unfavorable CO_2_^•**–**^ intermediate, proton-assisted multiple-electron reductive products including chemicals and/or hydrocarbon are commonly obtained so as to lower the activation energy of photocatalytic CO_2_ conversion [10]. Even so, the formation of high-order proton- and electron-transferring products still needs to surmount a considerable kinetic barrier, and the competitive H_2_ evolution further increases the difficulty for getting the aimed product selectively [13,14]. For instance, the photosynthesis of CH_4_—one kind of the most desirable and valuable hydrocarbon fuels in the photoreaction system—has been a great challenge [15], since the accomplishment of the eight-electron-transport process requires the photocatalyst to offer both strong reducing capability and sufficient electrons theoretically.

At present, the reported photocatalysts for CH_4_ formation mainly fall into two categories: (i) heterogeneous nanoscale semiconductors [16–18], which exhibit relatively low activity and selectivity for CH_4_ despite suitable band gaps and good recyclability; and (ii) homogeneous molecular metal complexes, with identifiable active centers, very few of which show high activity and selectivity for CH_4_ [19]. Although their crystallography essence is propitious to the clarification of the photocatalytic mechanism [20–26], the structural dissolubility makes them hard to separate from reductive products. In view of the practical application, constructing a heterogeneous photocatalyst with a well-defined structure for selective CH_4_ generation may be a good chance. Recently, a handful of moderate- or high-valence metal or metal cluster-based coordination frameworks (MCFs) have been investigated in the field of CO_2_ photoconversion [27–37], but the majority of reductive products primarily focus on two-electron transferred CO/HCOOH. Only the individual porphyrin-based MCF family (MOF-525) presents a low selectivity for CH_4_ generation [28], which is probably related to the insufficient redox and electron-donating abilities of catalytically active units. In addition, the structural collapsibility of ordinary MCFs in aqueous phase enables the photocatalytic reaction to occur exclusively in an organic medium [29,30] that no doubt exerts adverse effects on the environment.

Based on the aforementioned considerations, we conceived that polyoxometalate (POM)-based coordination frameworks (POMCFs), with well-known structural stability and favorable catalytic performance [38,39], are probably more beneficial to execute the photocatalytic reduction of CO_2_ due to the synergistic effect originated from the integration of POM and MCF [40–43]. In particular, the Zn-ϵ-Keggin cluster of the PMo_12_ ‘electron sponges’ family [44,45], including eight Mo^V^ atoms, can behave as a strong reductive component and contribute eight electrons theoretically. In addition, the Zn-ϵ-Keggin, a tetrahedral node, is formed by four-trapped Zn (II) locating in ϵ-Keggin (PMo_12_). Compared with most anionic POMs, the ϵ-Keggin modified with metal Zn becomes a cationic cluster, which is favorable for coordination with organic ligands. Consequently, if the reductive POM cluster and porphyrin derivative can be employed to fabricate POMCF, having both the visible-light-harvesting and photo-excited electron migration, which would be a good strategy towards selectively photoreducing CO_2_ to multielectron-reductive products.

Herein, we report on two structural analogous porphyrin-based POMCFs, [PMo^V^_8_Mo^VI^_4_O_35_(OH)_5_Zn_4_]_2_[Zn-TCPP][2H_2_O]·xGuest (**NNU-13**) and [PMo^V^_8_Mo^VI^_4_O_35_(OH)_5_Zn_4_]_2_[Zn-TCPP][2H_2_O]·yGuest (**NNU-14**), constructed with {ϵ-PMo^V^_8_Mo^VI^_4_O_40_Zn_4_} (Zn-ϵ-Keggin [45], PMo_12_ capped with four Zn^II^ ions) nodes and porphyrin derivative (H_2_TCPP) bridging ligands. Both of them show expected architectural stability and preeminent performance on the photocatalytic reduction of CO_2_. The whole photocatalytic reaction was performed in aqueous solution, without any participation of a photosensitizer and precious-metal co-catalyst. It is worth noting that the strong reducing ability of the Zn-ϵ-Keggin entity, combined with the remarkable optical and electrical properties of the TCPP linker, successfully endows these compounds with superior photocatalytic selectivity for CH_4_ production. To our knowledge, the high CH_4_ selectivity of 96.6% for **NNU-13** has surpassed all of the reported heterogeneous coordination framework-based photocatalysts [46], while **NNU-14** displays a slightly low CH_4_ selectivity of 96.2% by reason of its subtle structural deformation.

## RESULTS AND DISCUSSION

**Figure 1.**
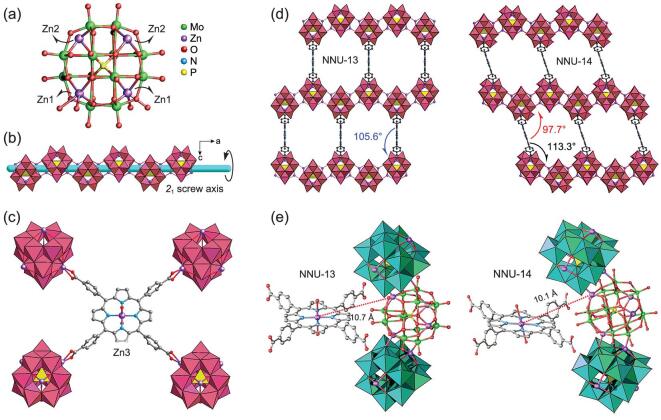
Ball-and-stick view of **NNU-13** and **NNU-14** crystal structures. (a) Four Zn atoms tetrahedron-capped Zn-ϵ-Keggin cluster; (b) a wavy POM chain was formed by Zn-ϵ-Keggin units, under the symmetry operation of the *2*_1_ screw axis; (c) four carboxyl groups of every Zn-TCPP ligand concurrently in contact with four different Zn-ϵ-Keggin clusters from four POM chains; the dihedral angles between the POM chain and Zn-TCPP metalloligand in (d) **NNU-13** and **NNU-14** (coordinated H_2_O molecules have been eliminated for clarity); the shortest Zn–Zn3 distances in (e) **NNU-13** and **NNU-14**. Color code: Mo, green; Zn, purple; O, red; N, blue; P, yellow; C, grey. Hydrogen atoms have been eliminated for clarity.

Crystalline **NNU-13** and **NNU-14**, prepared through similar hydrothermal synthesis protocols (Supplementary Fig. 1), show nearly identical host frameworks constructed from Zn-ϵ-Keggin nodes and four-connected TCPP linkers. Single-crystal X-ray diffraction analysis demonstrated that these two compounds crystallize in the orthorhombic *Fmmm* (**NNU-13**) and monoclinic *C2/m* (**NNU-14**) space groups (Supplementary Table 1), respectively. We have been working on the polyoxometalate-grafted photosensitive/electrosensitive ligands, such as **NNU-13** and Co-PMOF [47]. Compared to the synthesis method of **NNU-13**, the pH value of the solution was different and tris-(4-pyridyl)triazine (TPT) was additionally added when **NNU-14** was synthesized. The TPT acts as a templating agent in the synthesis of **NNU-14**. In each Zn-ϵ-Keggin cluster, two Zn1 sites along the *b* axis coordinate with two different TCPP ligands, while the remaining two Zn2 sites along the *a* axis oppositely bridge with two adjacent Zn-ϵ-Keggin motifs, under the symmetry operation of the *2*_1_ screw axis, forming a wavy POM chain in the end (Fig. 1a and b). In comparison, the octahedral Zn3 atom is captured by the ‘pocket’ of the TCPP center with two axial coordinated H_2_O molecules. Four carboxyl groups of each TCPP ligand concurrently are linked to four Zn1 atoms from different Zn-ϵ-Keggin clusters of four POM chains (Fig. 1c). Subsequently, a 3D network is established by connecting Zn-TCPP linkers with Zn1 sites of the POM chain alternately (Supplementary Figs 2 and 3), leading to an approximate dihedral angle of 105.6° between the POM chain and Zn3-TCPP metalloligand along the crystallographic *a* axis (Fig. 1d). Ultimately, **NNU-13** shows a two-fold interpenetrated ***mog*** topological structure (Supplementary Fig. 4a) as a result of multiple existing symmetry elements (rotation and screw axes together with mirror and glide planes). Likewise, **NNU-14** also has the same ***mog*** topology (Supplementary Fig. 4b), but its comparatively low spatial symmetry gives rise to approximate dihedral angles of 113.3° and 97.7° between the POM chain and Zn-TCPP linker along the crystallographic *a* axis (Fig. 1d). Notably, this structural distortion leads to the divergence on lattice parameters (Supplementary Fig. 5) and the shortest Zn–Zn distances (between the active Zn center in the TCPP pocket and the nearest Zn atom on the Zn-ϵ-Keggin cluster) of 10.7 (**NNU-13**) and 10.1 Å (**NNU-14**), respectively (Fig. 1e and Supplementary Table 2b).

The phase purity and chemical stability of **NNU-13** and **NNU-14** compounds were confirmed by powder X-ray diffraction (PXRD) patterns (Supplementary Fig. 6). The two POMCF samples were separately immersed into aqueous solutions with various pH values from 5 to 12 for at least 12 h. Noticeably, the PXRD patterns of all treated crystals remain intact, indicating that no phase transition or structural collapse occurred, even under the condition of the conventionally artificial photosynthesis-reaction system (Supplementary Fig. 6) [48]. The two POMCFs are relatively stable, mainly due to the high-temperature hydrothermal synthesis and small pore sizes. There are many other strategies that can be used for the synthesis of stable Zn-MOF such as the introduction of hydrophobic methyl groups [49]. **NNU-13** can adsorb CO_2_ with the maximum uptake of 34.1 cm^3^/g at 298 K, while **NNU-14** can adsorb CO_2_ with the maximum uptake of 14.3 cm^3^/g at 298 K (Supplementary Fig. 7). Both **NNU-13** and **NNU-14** have the ability to capture CO_2_, which is conducive to CO_2_ photoreduction. Besides, in order to validate the visible-light-enrichment capability, the corresponding diffuse reflectance UV-vis spectra were characterized. Both the compounds **NNU-13** and **NNU-14** exhibit broad adsorption covering the whole UV-vis region, as well as a red shift of the Soret band, along with a slight increase in the band intensity (Supplementary Fig. 8a). Moreover, the decreased number of adsorption peaks at the Q band compared with the free H_2_TCPP ligand, associated with increased molecular symmetry for metalloporphyrin, indicates the occurrence of charge transfer between the zinc ion and the TCPP ligand. Based on these results, the band gaps are ∼1.59 (**NNU-13**) and 1.64 eV (**NNU-14**)—slightly higher than the H_2_TCPP ligand of 1.53 eV (Supplementary Fig. 8b)—unveiling the potential for these POMCFs being as semiconducting photocatalysts. To elucidate the semiconductor character of title compounds and their possibilities for the subsequent photocatalytic conversion of CO_2_, Mott−Schottky measurements were performed at different frequencies (Supplementary Fig. 8c and d). From the C^−2^ values versus the applied potentials function relationship, the positive slopes of the curves are consistent with that of the typical n-type semiconductors, and the flat-band potentials determined from the intersection point are approximately −1.18 V versus Ag/AgCl (**NNU-13**) and −1.30 V versus Ag/AgCl (**NNU-14**). Since the bottom of the conduction band (LUMO) in the n-type semiconductors is commonly close to the flat-band potential [50], the LUMO of **NNU-13** and **NNU-14** can be estimated to be −0.98 V (versus NHE) and −1.10 V (versus NHE), respectively (Supplementary Fig. 8c and d). In view of such negative potentials for LUMO in POMCFs, the CO_2_ molecule theoretically can be converted into CO (−0.53 V versus NHE) and CH_4_ (−0.24 V versus NHE) upon visible-light irradiation. The CV curves of **NNU-13** and **NNU-14** were carried out in the reaction solution and four pairs of redox peaks appeared in the potential range from −0.6 to 0.5 V versus Ag/AgCl, attributed to the four-single-electron transfer of the POM secondary building block in the alkaline solution (Supplementary Fig. 9).

**Figure 2.**
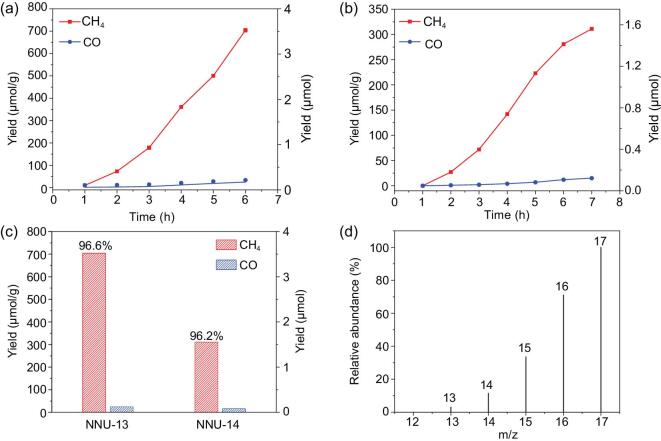
Performance for photoreduction of CO_2_ into CH_4_. Amounts of CH_4_ and CO produced as a function of the visible illumination time over (a) **NNU-13** and (b) **NNU-14**; (c) the total product yield and selectivity of gas products in the photoreduction of CO_2_; (d) the mass spectra of ^13^CH_4_ recorded under a ^13^CO_2_ atmosphere, and the detail spectra of the comparative experiments are shown in supplementary information. The photoreduction of the CO_2_ reaction occurred in the H_2_O/TEOA (14:1 V/V, 30 mL) solution with photocatalyst (5 mg) and illuminated via a Xe arc lamp with a UV-cutoff filter (420–800 nm).

The visible-light-driven CO_2_ reductions of **NNU-13** and **NNU-14** were conducted under a pure CO_2_ atmosphere in aqueous solution with triethanolamine (TEOA) as a sacrificial agent, in the absence of any photosensitizer and precious-metal co-catalysts. Gaseous CH_4_ and CO are the main reactive products detected by gas chromatography and no competitive H_2_ evolution was observed in the whole photoreaction process (Fig. 2 and Supplementary Figs 10 and 11). Such low-photocatalytic H_2_-production activities of **NNU-13** and **NNU-14** can be further supported by replacing CO_2_ with N_2_ (Supplementary Table 3). After 6 h, only 0.055 (**NNU-13**) and 0.035 (**NNU-14**) μmol H_2_ were detected under identical reaction conditions, without any CO generated. As indicated by Fig. 2a and b, the output of CH_4_ increases steadily with a prolonged time of light irradiation, while the CO yield has a negligible growth. With the reaction going on, the amount of CH_4_ for **NNU-13** reaches a maximum of 3.52 μmol (i.e. 704 μmol g^−1^) after 6 h, whereas **NNU-14** performs a CH_4_ generation of 1.56 μmol (i.e. 312 μmol g^−1^) after 7 h. By contrast, the CO amounts determined after the reaction peaked at 4.2 μmol g^−1^ h^−1^ (**NNU-13**) and 1.2 μmol g^−1^ h^−1^ (**NNU-14**). Moreover, both **NNU-13** and **NNU-14** exhibit high selectivity (CH_4_ over CO) of 96.6% and 96.2% (Fig. 2c), due to the similar connection strategy between the POM and TCPP. It is worth noting that both the selectivity and the activity of CH_4_ for these POMCFs are the highest among the reported heterogeneous coordination framework-based photocatalysts applied in the photocatalytic reduction of CO_2_ (Supplementary Table 4) [28,34]. There are no Co and Fe in **NNU-13** and **NNU-14**, which reveals that the generation of CH_4_ eliminates the interference of Fe and Co (Supplementary Table 5). The relevant parameters of this photoreduction system, including TONs, TOFs and Φ CH_4_, are summarized in Supplementary Table 6. The overall CH_4_-TON of **NNU-13** was estimated to be 3.56, which is higher than that of **NNU-14** (1.57), suggesting that both of these complexes are indeed catalytic for CO_2_-to-CH_4_ conversion. The apparent quantum yield of these optimized POMCFs-promoted CO_2_-photoreduction systems were estimated to be 0.04% (**NNU-13**) and 0.02% (**NNU-14**) at a monochromatic irradiation of λ = 550 nm. Obviously, the compound **NNU-13** has higher photocatalytic CO_2_-reduction activity than **NNU-14**, as demonstrated by evidently distinguishing the transient photocurrent responses and electrochemical-impedance spectra (Supplementary Figs 12 and 13). Although both of them display obvious photocurrent responses to visible-light irradiation, **NNU-13** has a much higher photocurrent than that of **NNU-14**, which means a better separation efficiency of the photo-induced electron–hole pairs for **NNU-13**. At the same time, the semicircle size of the Nyquist plot for **NNU-13** is much smaller, reflecting an acceleration of the interfacial charge-transfer process, in accordance with the above photocurrent-response results. These differences in the charge separation and the kinetics of charge transfer can be attributed to the structural distortion on the dihedral angles between the POM chain and the Zn-TCPP linker in **NNU-13** and **NNU-14**.

Additionally, in a series of reference experiments that were conducted in the absence of POMCF catalysts, CO_2_ or light illumination, no detectable products were observed in the reaction system (Supplementary Table 3). Moreover, the influence of different POMCF qualities on the activity of the photoconversion CO_2_ reaction was also investigated [31], for which the high selectivity of CH_4_ over CO was still retained (Supplementary Fig. 14). The photocatalytic stability of these POMCFs was evaluated using recycling tests. From the time-course plots of CH_4_ evolution, the photocatalysts maintain excellent activities even after three cycles (Supplementary Fig. 15). The slight decay in the CH_4_-evolution rate in each successive run is probably attributed to the mass loss in the recovery process of used samples. There has been no noticeable alteration on the infrared spectra and PXRD patterns performed before and after three cycles of the photocatalytic reaction that confirmed the structural robustness of **NNU-13** and **NNU-14** (Supplementary Figs 16 and 17). Moreover, the filtrate reaction was further executed to confirm the heterogeneous nature of these POMCFs. Once the catalysts were removed from the reaction system after several hours of photocatalytic reduction, the production of CH_4_ and CO stopped, which clearly precluded the influence of potential intermediate or decomposed active components on the product generation (Supplementary Fig. 18). From these results, both **NNU-13** and **NNU-14** possess high durability toward the CO_2_ photocatalytic reduction under visible-light irradiation. After the photocatalytic reaction, extremely trace amounts of formic acid was produced in aqueous solution as detected by ion chromatography. An isotopic experiment using ^13^CO_2_ as substrate was performed under identical photocatalytic reaction conditions to validate the carbon source of the generated gases [18,33,51] and the products were analysed by gas chromatography and mass spectra. As shown in Fig. 2d and Supplementary Figs 19 and 20, the peaks at m/z = 17/16/15/14 and m/z = 29 were assigned to fragments of ^13^CH_4_ and ^13^CO, respectively, providing solid proof that these POMCFs are indeed active and capable of selectively converting CO_2_ to CH_4_ under visible-light irradiation. In addition, taking **NNU-13**, for example, the experiment under the CO atmosphere was carried out and the result verified that CO is an intermediate for further CH_4_ generation (Supplementary Fig. 21).

To disclose the origin of and difference in the photocatalytic performances of **NNU-13** and **NNU-14**, the roles of the structural components including the POM subunit (Zn-ϵ-Keggin) and TCPP organic linker were considered. On the one hand, it is well known that all the previously reported TCPP-based MOF photocatalysts in the photocatalytic CO_2_-reduction system, involving mono/multi-metal active center either in a moderate- or high-valence state, tend to produce high selectivity and activity, for CO over CH_4_, such as the MOF-525 family and Zr-MOFs [28,34]. Although high-valent metal clusters could serve as a multielectron acceptor in some cases, its poor reducing ability makes it hard to contribute enough electrons to satisfy the eight-electron process of CH_4_. Therefore, based on the reported porphyrin-MOFs for CO_2_ photoreduction, two-electron transferred CO as a frequently predominant gaseous product is probably related to the reduction intensity of the active center. To confirm the photocatalytic activity of **NNU-13** in the production of CH_4_, a series of control experiments were carried out, which involved Zn-ϵ-Keggin (Supplementary Figs 22 and 23), Zn-TCPP and the mixture of Zn-ϵ-Keggin and Zn-TCPP as independent photocatalysts (Supplementary Table 7). It is obvious that both the Zn-ϵ-keggin cluster and the Zn-TCPP linker produced CO (370 and 16 μmol g^−1^, respectively), while the mixtures of Zn-ϵ-Keggin (POM) and Zn-TCPP (sensitizer) produced CO and CH_4_ with a lower yield and selectivity for CH_4_. By contrast, **NNU-13** assembled with POM and porphyrin showed a higher yield and selectivity for CH_4_. Moreover, the generated CO from Zn-ϵ-Keggin was mainly used for CH_4_ formation. Therefore, the integration of photosensitive TCPP with reductive Zn-ϵ-Keggin within **NNU-13** and **NNU-14** played an indispensable role in obtaining high CH_4_ selectivity. On the other hand, in order to eliminate the potential impact of the TCPP linker on high CH_4_ selectivity and the activity of **NNU-13** and **NNU-14**, similar Zn-ϵ-Keggin-based POMCF (**NNU-12**) (Supplementary Figs 24 and 25) [52], with H_2_BCPT instead of TCPP as the bridging ligand and light-harvesting unit, was synthesized to execute a comparable CO_2_ photocatalytic reaction. We found that **NNU-12** still maintained a high CH_4_ over CO selectivity of 81.3% (Supplementary Table 8), which again corroborated the significance of the POM component with a strong reducing ability and electron-transferring ability (i.e. redox ability). Because of the eight Mo^V^ atoms in the reductive Zn-ϵ-Keggin cluster, it can theoretically easily offer eight electrons to complete the eight-electron reduction process from CO_2_ to CH_4_. Of course, the relatively poor photocatalytic activity of CH_4_ in **NNU-12** also indicated the important roles of the excellent visible-light-harvesting and electron-transport capabilities of the TCPP linker, in particular for **NNU-13** and **NNU-14**, whose high photocatalytic properties are primarily realized by the synergistic incorporation of the advantages of the Zn-ϵ-Keggin node and TCPP linker. In other words, the photo-generated electrons transferred from the TCPP linker flowing to the POM cluster are indispensable to the efficient photocatalytic CO_2_ reduction. Additionally, the relative location and distance between the active center in the TCPP pocket and the Zn-ϵ-Keggin cluster (Fig. 1e) result in different levels of competition on the CO_2_ molecule absorbed, as well as different numbers of active sites in the crystal cells (Supplementary Fig. 5) of **NNU-13** and **NNU-14**, both of which are probably responsible for the discrepant photocatalytic activities for these POMCFs.

**Figure 3.**
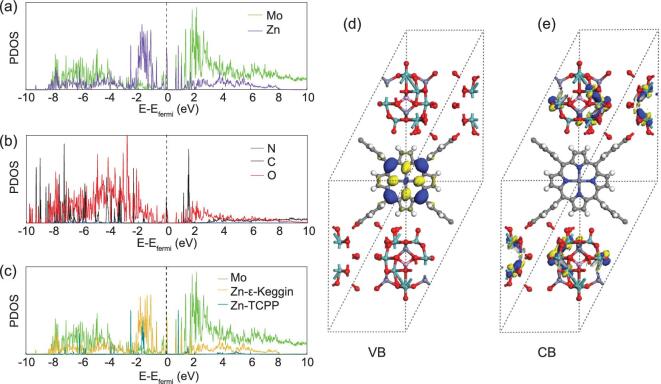
Partial density of states (PDOS). (a) Mo and total Zn atoms, (b) C, O and N atoms, (c) Mo atoms, Zn atoms in POMs and Zn atoms in TCPP. Partial-charge-density maps of (d) valence band and (e) conduction band above the Fermi level for **NNU-13**.

To obtain a deeper understanding on the photo-excited charge-carrier separation mechanism in these porphyrin-based POMCF catalytic systems, theoretical calculation was carried out based on density functional theory (DFT). The total density of states (TDOS), partial density of states (PDOS) and band structures are shown in Supplementary Figs 26 and 27 and 3a–c, and the Fermi levels were taken as the energy zero. From the TDOS, we could see that the alpha-DOS (DOS: density of state) is different from the beta-DOS, which suggested that the MCF was with a single electron and a magnetic property (Supplementary Fig. 26). From the PDOS, we could see that the tops of the valence band (VB) were composed of the states of all elements, which means the top of the VB was distribution by both POMs and TCPP. The bottom of the conduction band (CB) was composed of the O 2p states, Zn 3d states and Mo 4d states. We could see that the Zn 3d states that composed the bottom of the CB are those in the POMs. It suggested that the bottom of the CB was distributed substantially by the POMs. This composition of the DOS suggested that both the POMs and the TCPP could act as electron donors while only POMs act as electron acceptors. In this case, once the light irradiates the MCF, the electrons in the VB are exited. Despite the charge transfer in the POMs, the electrons in the TCPP will be excited and transferred to the POMs, which matches our experimental prediction very well. In order to show the distribution clearly, the partial-charge-density maps of the VB and CB are shown in Fig. 3d and e. The partial-charge-density maps were intuitional figures involving the predominant distribution and transferring direction of CB and VB electrons. According to the partial-charge-density maps, we could see that the VB was distributed by the TCPP while the CB was distributed by POMs. Despite the charge transfer in POMs, the TCPP may act as an electron donor and POMs may act as electron acceptors, which matched well with our analysis of DOS. Based on the above calculations, the mechanism could be explained as follows: the light irradiates on the MCF and the electrons in the VB are first excited. Subsequently, the electrons in the VB (TCPP) transfer to the CB (POMs), which can be further utilized for the activation and reduction of absorbed CO_2_.

**Figure 4.**
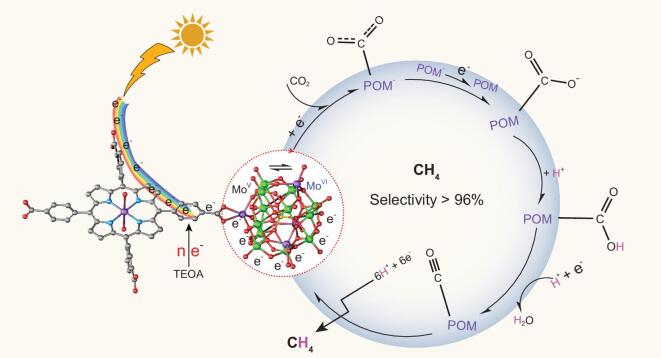
Proposed mechanism for photocatalytic CO_2_ reduction over POMCFs under visible-light irradiation.

In light of the combination of the experimental results and theoretical calculations, a mechanism with respect to the superior photocatalytic CO_2_-reduction performances of TCPP-based POMCFs and possible photo-generated electron-transport pathway in the system was proposed (Fig. 4). For most of the porphyrin-based MCFs, the catalytic active sites generally situated at the metal-modified porphyrin center and/or relatively high-valent metal/metal cluster nodes [28,34]. The porphyrin segments absorb the photos to generate electrons and allow the procreant electron transfer to the central metal ions/cluster, leading to the formation of CO as the primary gas product. However, for the TCPP-based POMCFs with strong reducing ability (**NNU-13** and **NNU-14**), the predominant product is CH_4_. As the calculations revealed, the electrons of the tops of the VB were mainly concentrated on the TCPP ligands (Fig. 3d). The TCPP linkers absorb the light and move the photo-generated electrons on the VB toward the reductive Zn-ϵ-Keggin fragments. Zn-ϵ-Keggin and Zn-TCPP have been used as catalysts to perform CO_2_ photoreduction reactions, respectively, and the results are listed in Supplementary Table 7. Obviously, these results illustrate that the synergistic effect between the Zn-ϵ-Keggin and the Zn-TCPP in **NNU-13** can further reduce the produced CO to CH_4_, and CO is indeed the intermediate for CO_2_-to-CH_4_ conversion. Ultimately, the enrichment of electrons on the reductive Zn-ϵ-Keggin fragments (based on the eight Mo^V^ atoms) probably makes it preferable to provide enough electrons to CO_2_, allowing the further combination with the protons from the water to produce eight-electron-transferred CH_4_. In the meantime, trace amounts of two-electron-transferred CO are still more likely to be generated on the Zn-TCPP sites relying on the ligand-to-metal charge transition (LMCT) effect. Therefore, CH_4_ was the predominant reduced product with high selectivity in the gas production of the TCPP-based POMCF catalytic system. Of course, the Mo^VI^ ions also receive the generated electrons and then deliver them, but with lower activity than Mo^V^ atoms. The TEOA as the sacrificial reagent consumed the electron holes of the VB. The protons mainly come from the water due to its being the reaction solvent. An additional verification experiment using **NNU-13** and acetonitrile (aprotic solvent including a little water) as photocatalyst and reagent was performed to prove this consideration, only generating very small amounts of CO (∼10 μmol g^−1^).

## CONCLUSION

In summary, the heterogeneous photocatalytic reduction of CO_2_ to CH_4_ in the aqueous phase was realized for the first time over POM-containing MCFs, **NNU-13** and **NNU-14**, fabricated with reductive Zn-ϵ-Keggin cluster and visible-light-responsive TCPP linker. Theoretical calculations revealed that the photo-generated carriers of the VB and the CB are mostly distributed on the TCPP group and Zn-ϵ-Keggin cluster, respectively. Consequently, the photo-excited electrons more easily flow to the POM port by efficient intercoupling between the reductive Zn-ϵ-Keggin unit and the TCPP linker. Therefore, these POMCFs exhibit high photocatalytic CH_4_ selectivity (>96%) and activity that have far surpassed all of the reported MCF-based photocatalysts. Note that the introduction of POM building blocks with potent reducing ability not only endows **NNU-13** and **NNU-14** with favorable structural rigidity, but also it indeed facilitates the photocatalytic selectivity of CH_4_ by theoretically delivering adequate electrons to accomplish the eight-electron reduction of the CO_2_ molecule. We expect that such a feasible approach, assembling a strong reducing component into visible-light-sensitized photocatalyst architecture, can ignite research enthusiasm towards the construction of efficient POMCF photocatalysts for the highly selective reduction of CO_2_ to CH_4_ or other high-valued hydrocarbons.

## METHODS

### Synthesis of NNU-13

Na_2_MoO_4_·2H_2_O (310 mg, 1.28 mmol), H_3_PO_3_ (10 mg, 0.125 mmol), zinc chloride (68 mg, 0.50 mmol), tetrabutylammonium hydroxide (TBAOH) 10 wt % solution in water (250 μL) and H_2_O (3.5 mL) were charged in a Pyrex vial and stirred for 10 min. Then, the pH value of the mixture was adjusted to 5.0 by 2 mol/L HCl. Subsequently, meso-tetra(4-carboxyphenyl) porphine (H_2_TCPP) (118.23 mg, 0.15 mmol), Mo powder 99.99% (25 mg, 0.26 mmol) and 250 μL dimethylacetamide were added into the mixture and stirred for 15 min. The mixture was sealed in a 15-mL Teflon-lined stainless-steel container and then heated at 180°C for 72 h. After cooling to room temperature at 15°C h^−1^, dark-violet block crystals of **NNU-13** were collected (yield 76% based on H_2_TCPP).

### Synthesis of NNU-14

Na_2_MoO_4_·2H_2_O (618 mg, 2.55 mmol), H_3_PO_3_ (20 mg, 0.25 mmol), zinc chloride (136 mg, 1.00 mmol), tetrabutylammonium hydroxide (TBAOH) 10 wt % solution in water (500 μL) and H_2_O (7 mL) were charged in a Pyrex vial and stirred for 10 min. Then, the pH value of the mixture was adjusted to 2.0 by 2 mol/L HCl. Subsequently, tris-(4-pyridyl) triazine (TPT) (46.85 mg, 0.15 mmol), Meso-Tetra(4-carboxyphenyl) porphine (H_2_TCPP) (118.23 mg, 0.15 mmol), Mo powder 99.99% (25 mg, 0.26 mmol) and 500 μL dimethylacetamide were added into the mixture and stirred for 15 min. The mixture was sealed in a 15-mL Teflon-lined stainless-steel container and then heated at 180°C for 72 h. After cooling to room temperature at 15°C h^−1^, dark-violet cuboid crystals of **NNU-14** were obtained (yield 64% based on H_2_TCPP).

### Single-crystal X-ray crystallography

Single-crystal X-ray diffraction (XRD) data of **NNU-13** and **NNU-14** have been measured on a Bruker APEXII CCD diffractometer with graphite-monochromated Mo Kα radiation (λ = 0.71073 Å) at 296 K. These two structures were solved by a direct method and the SHELXT program, and refined on Olex^2^ software by the SHELXL program. A multi-scan technique was used for Absorption corrections. The TBA^+^ cations could not be detected in the structure, and some other guest molecules are disordered. Thus, the data were further corrected with SQUEEZE on the PLATON software to eliminate some guest molecules and then check the space group of these two crystals. The detailed crystallographic information and selected bond lengths are listed in Supplementary Tables 1 and 2a.

### Photochemical measurements

The photocatalytic CO_2_-reduction experiments were probed on an evaluation system (CEL-SPH2N, CEAULIGHT, China) in a 100-mL quartz container, and the two round openings of the container were sealed with rubber mats. A xenon arc lamp (CEL-HXF300/CEL-HXUV300, 200 mW/cm^2^) with a UV-cutoff filter (420–800 nm) was utilized as irradiation source. The as-prepared photocatalyst (5 mg) was evacuated in mixed solutions (30 mL) with analytical reagent (AR) TEOA (2 mL) and deionized water (28 mL) and pre-degassed with CO_2_ (99.999%) for 30 min to remove air before irradiation. The pH values were determined to be 10.5, 10.5 and 7.3 in the absence of the catalyst with the TEOA, after suspension of the catalyst and after sparging with CO_2_ (30 min). The sealed reaction system (CO_2_ pressure of 1 atm) was positioned about 10 cm away from visible-light source, and stirring was continued constantly to ensure that the photocatalyst particles were in suspension. The reactor was connected using a circulating cooling water system to maintain the temperature of the solution at around 20°C. Gaseous product was measured using gas chromatography (GC-7900, CEAULIGHT, China, column type: TDX-1) equipped with a flame ionization detector (FID) and a thermal-conductivity detector (TCD). The isotope-labeled experiments were recorded using gas chromatography-mass spectrometry (GC-MS, 7890A and 5875C, Agilent). GC-MS column (19091P-Q04PT), gas (He as carrier), flow rate (1.4 mL/min, 5.2027 psi), oven ramping parameters (injector temperature: 250°C, column temperature is constant temperatures: 30°C).

### Photoelectrochemical studies

Five milligrams of crystal samples were ground and dispersed in 1 mL mixed solution of ethanol and water under sonication for 1 h. Then, 10 μL above the turbid liquid was daubed on indium-tin oxide (ITO) glass with a fixed circular area (diameter 6 cm) and the electrode was dried for about 24 h in a desiccator. The electrochemical experiments were performed on an electrochemical workstation (CHI 660e) in a standard three-electrode system. The Mott–Schottky tests were performed in 0.2 M Na_2_SO_4_ solution, using a catalyst/ITO electrode as the working electrode, an Ag/AgCl electrode as the reference electrode and a Pt plate as the counter electrode at different frequencies of 500, 1000 and 1500 Hz. The photocurrent tests were carried out in tris-HCl electrolyte (0.1 M) at a bias of 0.0 V using an Xe lamp as the light source. Electrochemical-impedance spectroscopy measurement was carried out on the electrochemical workstation (Bio-Logic, VSP) by applying an AC voltage with 10-mV amplitude in a frequency range from 1000 kHz to 100 mHz.

## Supplementary Material

nwz096_Supplemental_File
