# Supplementary material for: Multielectron transportation of polyoxometalate-grafted metalloporphyrin coordination frameworks for selective CO2-to-CH4 photoconversion
Source: Natl Sci Rev. 2019 Jul 16;7(1):53–63. doi: 10.1093/nsr/nwz096 (PMC8288839; doi:10.1093/nsr/nwz096)
Supplement: nwz096_Supplemental_File [file nwz096_supplemental_file.doc]

Supplementary Information

**Multielectron transportation of polyoxometalate-grafted metalloporphyrin coordination frameworks for selective CO_2_-to-CH_4_ photoconversion**

Qing Huang,^1, †^ Jiang Liu,^1, †^ Liang Feng,^3^ Qi Wang,^2^ Wei Guan,^2^ Long-Zhang Dong,^1^ Lei Zhang,^1^ Li-Kai Yan,^2^ Ya-Qian Lan ^1, *^and Hong-Cai Zhou^3^

*1 Jiangsu Collaborative Innovation Centre of Biomedical Functional Materials, Jiangsu Key Laboratory of New Power Batteries, School of Chemistry and Materials Science, Nanjing Normal University, Nanjing, 210023, P. R. China*

*2 Department of Chemistry, Northeast Normal University, Changchun 130024, P. R. China*

*3 Department of Chemistry, Texas A&M University, College Station, Texas 77843-3255, United States*

*^†^ These authors contributed equally*

**Corresponding authors Y.-Q. L. E-mail:* [*yqlan@njnu.edu.cn*](mailto:yqlan@njnu.edu.cn)

Table of Contents

**Experimental Section and Theoretical calculations** S3

Supplementary Figure 1: Images of crystals S5

Supplementary Figure 2: The coordination environments of Zn(II) centers in NNU-13 S5

Supplementary Figure 3: The coordination environments of Zn(II) centers in NNU-14 S6

Supplementary Figure 4: Topology arrays of NNU-13 and NNU-14 S6

Supplementary Figure 5: The unit cell difference between NNU-13 and NNU-14 S7

Supplementary Figure 6: pH stabilities were recorded by PXRD patterns S7

Supplementary Figure 7: CO_2_ adsorption isotherms S8

Supplementary Figure 8: UV–visible diffuse reflection spectra and Mott–Schottky plot S8

Supplementary Figure 9: CV curves S9

Supplementary Figure 10: GC calibration lines of gas standards S9

Supplementary Figure 11: Gas chromatogram detection for products S10

Supplementary Figure 12: Transient photocurrent/time profiles S10

Supplementary Figure 13: Nyquist plots of catalysts S11

Supplementary Figure 14: The effect of the amount of catalysts S11

Supplementary Figure 15: The number of cycles in the experiments S12

Supplementary Figure 16: The IR spectra before and after test S12

Supplementary Figure 17: The PXRD patterns after photoreduction of CO_2_ experiment S13

Supplementary Figure 18: The filtrate reaction S13

Supplementary Figure 19: The mass spectra of ^13^CO and the Mass spectra of ^12^CH_4_ S14

Supplementary Figure 20: The full scan of Mass spectra S14

Supplementary Figure 21: The experiment under CO atmosphere S15

Supplementary Figure 22: Summary of the structure of NENU-499 S15

Supplementary Figure 23: PXRD patterns of Zn-ε-Keggin (NENU-499) S16

Supplementary Figure 24: Summary of the structure of NNU-12 S16

Supplementary Figure 25: The PXRD pattern of NNU-12 S17

Supplementary Figure 26: Total density of states S17

Supplementary Figure 27: Band structure S17

Supplementary Table 1: Crystal data and structure refinements S18

Supplementary Table 2: Selected bond lengths and distances of selected atoms S19

Supplementary Table 3: Conditional exploration for photoreduction of CO_2_ S21

**Supplementary Table 4**: Comparisons of performance on CO_2_ photoreduction S22

Supplementary Table 5: ICP-MS characterization S25

Supplementary Table 6: TON, TOF and Φ CH_4_ S26

Supplementary Table 7: The control experiments S27

**Supplementary Table 8**: Gas product species and CH_4_ selectivity S28

**Experimental Section**

**Materials and measurements.**

All reagents and solvents were commercially available and used as received. FTIR spectra in the range of 4000–400 cm^-1^ were obtained *via* KBr pellets on a Bruker Tensor 27 FT/IR spectrophotometer. PXRD patterns of the samples were performed in the range 3-50^o^ on a D/max 2500VL/PC diffractometer with Cu Kα radiation (λ =1.5406 Å) at 298K. The optical absorption and diffused reflectance spectra were carried out on a Cary 5000 UV-Vis-NIR spectrophotometer (Viarian, USA) using BaSO_4_ as a reflectance standard.

**Synthesis of Zn-ε-Keggin (NENU-499) [**1**].** A mixture of sodium molybdate dihydrate (618 mg, 2.55 mmol), Mo powder 99.99% (50 mg, 0.52 mmol), H_3_PO_3_ (20 mg, 0.25 mmol), zinc chloride (136 mg, 1.00 mmol), tetrabutylammonium hydroxide 40 wt % solution in water (120 μL, 0.18 mmol), and H_2_O (8 mL) was stirred for 20 min, and the pH was acidified to 4.8 with diluted HCl (2 M). Then, 6- nitrobenzimidazole (82 g, 0.50 mmol) was added to the mixture, which was transferred and sealed in a 15 mL Teflon-lined stainless steel container and heated at 180 °C for 72 h. After cooling to room temperature at 10 °C·h^−1^, the dark-red crystals were harvested for PXRD (Supplementary Figure 20).

**Synthesis of**  **Zn-TCPP.** 5, 10, 15, 20-Tetrakis(4-methoxycarbonylphenyl)porphyrin (TMCP) (1.7 g, 2.0 mmol) , ZnCl_2_ (3.5 g, 25.6 mmol), and DMF (200 mL) were mixed and refluxed for 12 h. A large amount of water was added after cooling down to room temperature. The as-synthesized material was filtered and washed with water (600 ml) for six times. The fuchsia solid sample was obtained (yield 90% based on TMCP). Then, the obtained ester, MeOH (50 mL) and THF (50 mL) were mixed and stirred. 50 mL KOH solution (5.2 g, 93.9 mmol KOH was dissolved in 50 mL H_2_O) was added into the mixture and was refluxed for one night. After reaction, the organic solvents were evaporated. To make the solid fully dissolve, moderate water was added to the mixed solution and heated. 2 M HCl was used to acidify the obtained fuchsia homogeneous solution until no further fuchsia precipitate was generated. The solid was collected and washed with deionized water until the pH of filtrate reaches about 5. Finally, the solid sample was dried in vacuum at 60 ^o^C [2].

**Synthesis of NNU-12.** A mixture of Na_2_MoO_4_·2H_2_O (618 mg, 2.55 mmol), ZnCl_2_(136mg, 1.00 mmol), H_3_PO_3_ (20 mg, 0.25 mmol), tetrabutylammonium hydroxide 10 wt % solution in water (480 μL, 0.18 mmol), and H_2_O (7 mL) was stirred 10 min, then, the PH was adjust to 4.0 with 2 M HCl solution. Subsequently, Mo powder 99.99% (25 mg, 0.26 mmol), 3,5-bis(4’-carboxyl-phenyl)-1, 2, 4-triazol (H_2_BCPT) (92.72 mg, 0.30 mmol) and appropriate DMA were added into the mixture of PH 4.0. Finally the mixture was stirred 30 min and sealed in a 15 mL Teflon-lined reactor and heated at 180 °C for 3 d. After cooling to room temperature at 10 °C·h^−1^, black block crystals of NNU-12 were collected (63% yield based on H_2_BCPT).

**Theoretical calculations.** All of calculations were performed with Cambridge Serial Total Energy Package (CASTEP) [3] plane-wave code in Material Studio software based on density functional theory (DFT) [4, 5]. The generalized gradient approximation (GGA) in the scheme of the Perdew-Burke-Ernzerhof (PBE) [6] was employed as exchange-correlation functional. All calculations were done with acutoff energy of 400 eV and the Brillouin-zone was sampled using a 7 × 4 × 4 Monkhorst-Pack mesh. In order to describe the van der Waals (vdW) interaction of the system properly, TS method of DFT-D [7] was applied. The SCF tolerance was set to 10^-5^ eV and the Koelling-Hamon relativistic treatment was used for scalar relativistic corrections to heavy element.


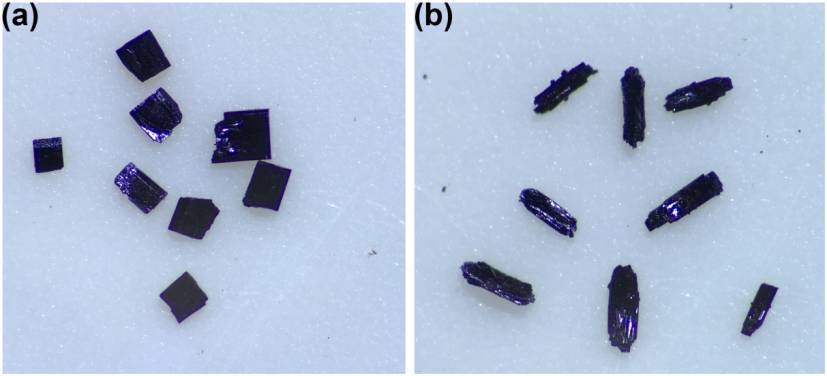


**Supplementary Figure 1** **|** The images of (a) **NNU-13** and (b) **NNU-14** under optical microscope.


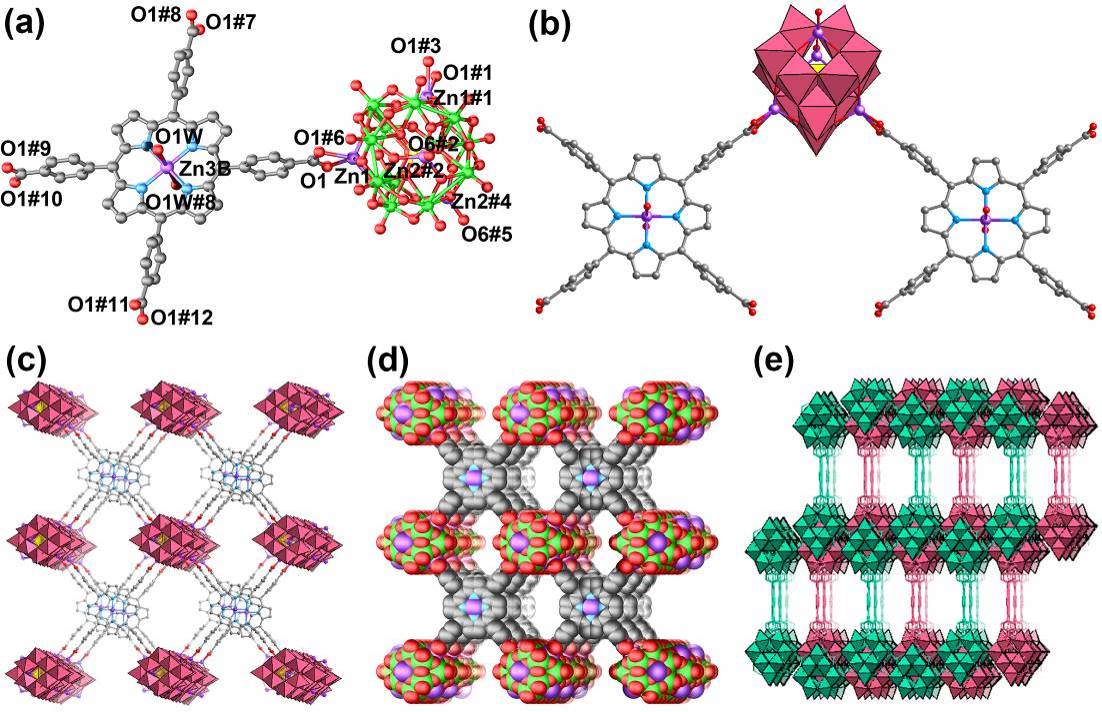


**Supplementary Figure 2** **|** Summary of the structural information of **NNU-13**: (a) The coordination environments of Zn(II) centers in **NNU-13**. Color code: C, black; N, blue; O, red; Zn, purple; Mo, green; P, yellow. Symmetry codes: #1 – *x*, *y*, *z*; #2 – *x*, 0.5 – *y*, 0.5 + *z*; #3 – *x*, *y*, 1 – *z*; #4 – *x*, 0.5 – *y*, 0.5 – *z*; #5 *x*, 0.5 – *y*, 0.5 – *z*; #6 *x*, *y*, 1 – *z*; #7 *x*, 1 – *y*, *z*; #8 *x*, 1– *y*, 1– *z*; #9 – 1 – *x*, 1 – *y*, 1 – *z*; #10 – 1 – *x*, 1 – *y*, *z*; #11 –1 – *x*, *y*, 1– *z*; #12 –1 – *x*, *y*, *z*. (b) Two trapped Zn1 atoms in Zn-ε-Keggin unit coordinate with two different Zn-TCPP metalloligands. (c) 3D (4, 4) connected framework was formed by the 4-connected TCPP linkers and POM chains. (d) 3D spacing stacking and (e) two-fold interpenetrated structure.


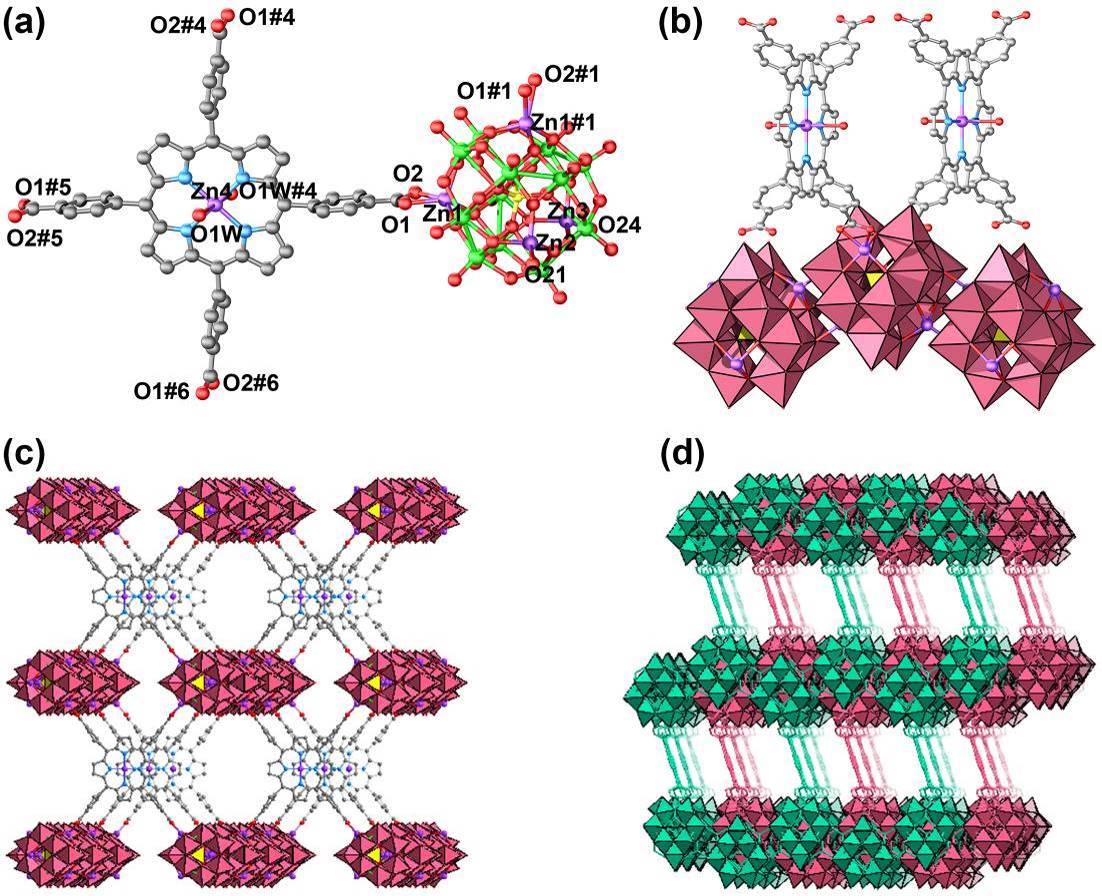


**Supplementary Figure 3** **|** Summary of the structural information of **NNU-14**: (a) coordination environments for the Zn(II) centers. Color code: C, black; N, blue; O, red; Zn, purple; Mo, green; P, yellow. Symmetry codes: #1 *x*, 1 – *y*, *z*; #2 1 – *x*, *y*, 1 – *z*; #3 – *x*, *y*, 1 – *z*; #4 1 – *x*, *y*, 2 – *z*; #5 1 – *x*, *y*, 2 – *z*; #6 *x*, – *y*, *z*. (b) Each Zn-ε-Keggin connected two Zn-ε-Keggin fragments and two TCPP liangds in **NNU-14**. (c) 3D frameworks formed by Zn-ε-Keggin fragments and TCPP linkers. (d) View along the *b* axis of the two-fold interpenetrated nets.


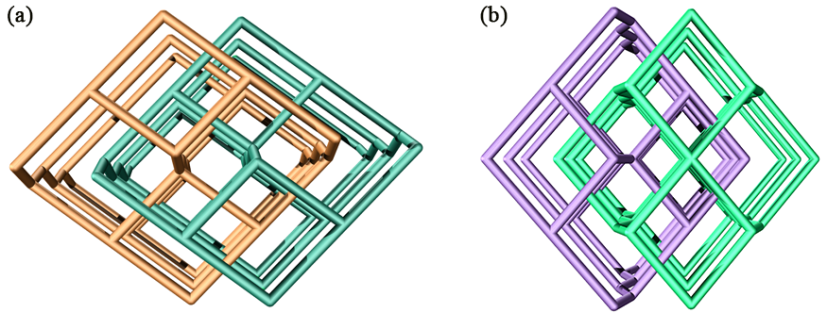


**Supplementary Figure 4** **|** Topology arrays of (a) **NNU-13** and (b) **NNU-14**.


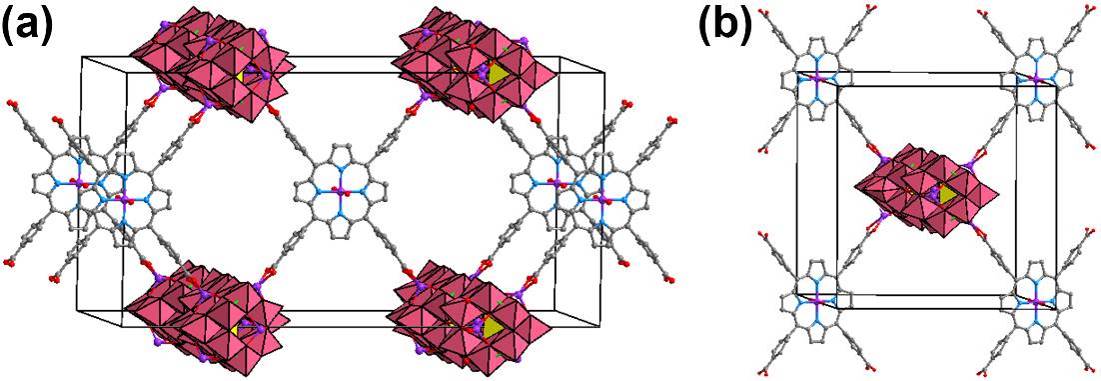


**Supplementary Figure 5** **|** The defference between **NNU-13** and **NNU-14**: (a) the contents of the unit cell for **NNU-13** are more than (b) the contents of unit cell for **NNU-14**. For clarity, just single framework was compared in unit cell.


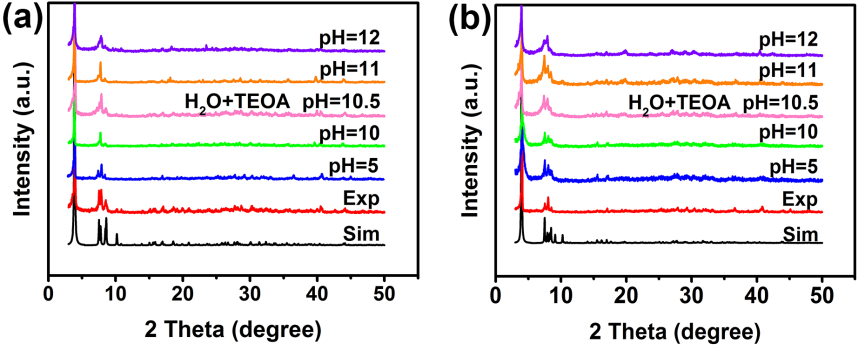


**Supplementary Figure 6** **|** PXRD patterns of (a) **NNU-13** and (b) **NNU-14** under different conditions for 12h, indicating their structural robustness upon harsh treatments. “Sim” represents the simulated pattern, and “Exp” represents the pattern of as-synthesized sample.


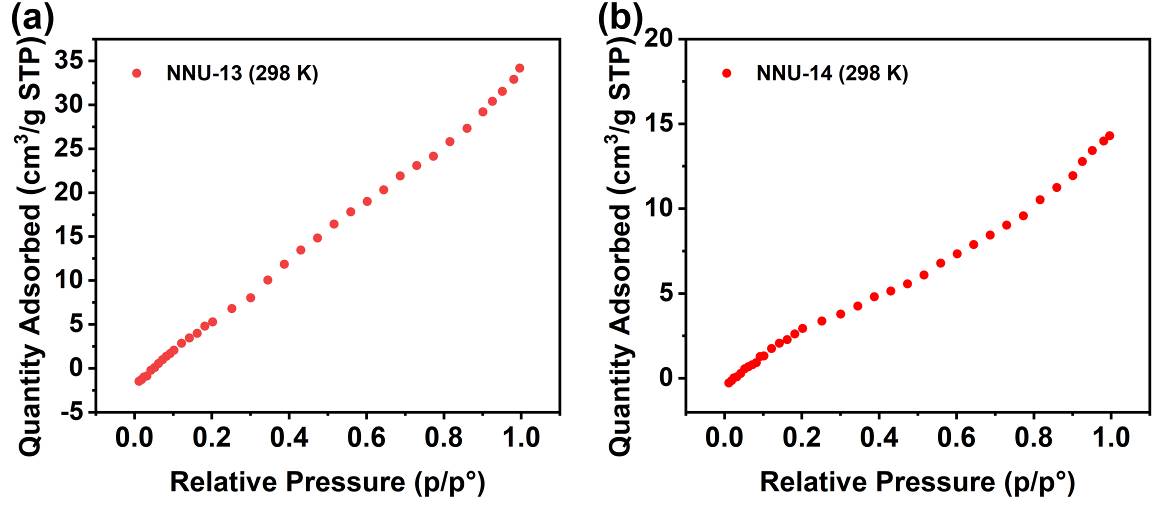


**Supplementary Figure 7 |** CO_2_ adsorption isotherms of (a) **NNU-13** and (b) **NNU-14** at 298 K.


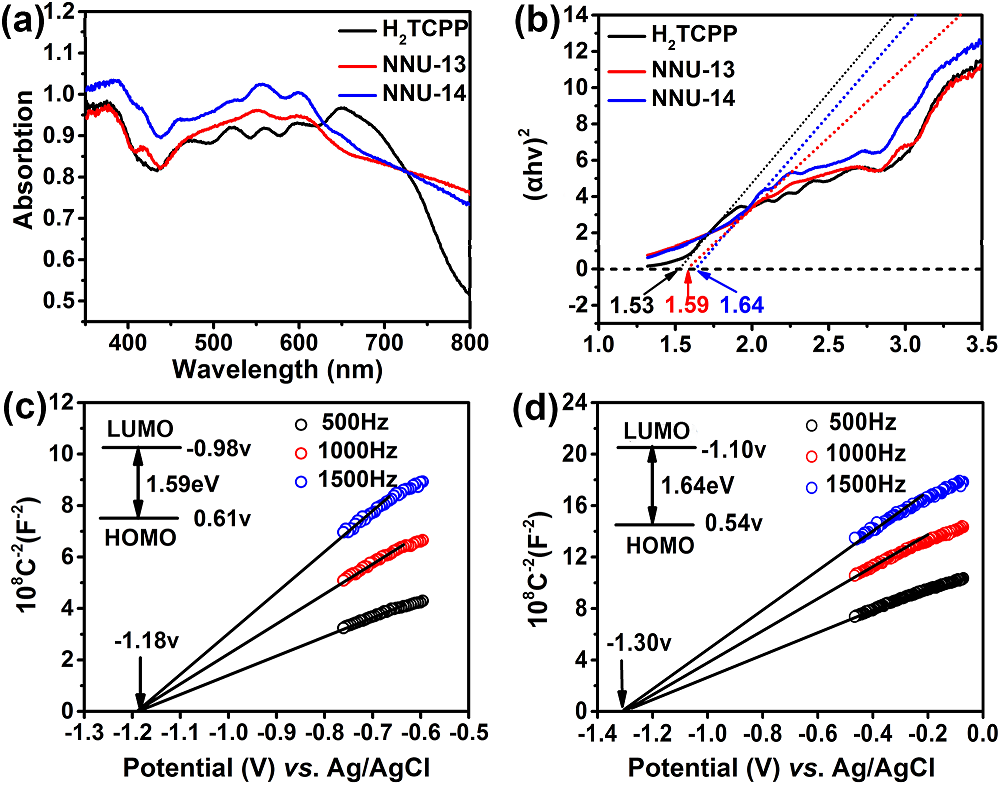


**Supplementary Figure 8** **|** (a) UV–visible diffuse reflection spectra and (b) the band gaps ((αhν)^2^ vs binding energy) calculated for **NNU-13**, **NNU-14** and H_2_TCPP samples. Mott–Schottky plots for (c) **NNU-13** and (d) **NNU-14** in 0.2 M Na_2_SO_4_ aqueous solution (with inset of the energy diagram of its highest occupied molecular orbital (HOMO) and lowest unoccupied molecular orbital (LUMO) levels.


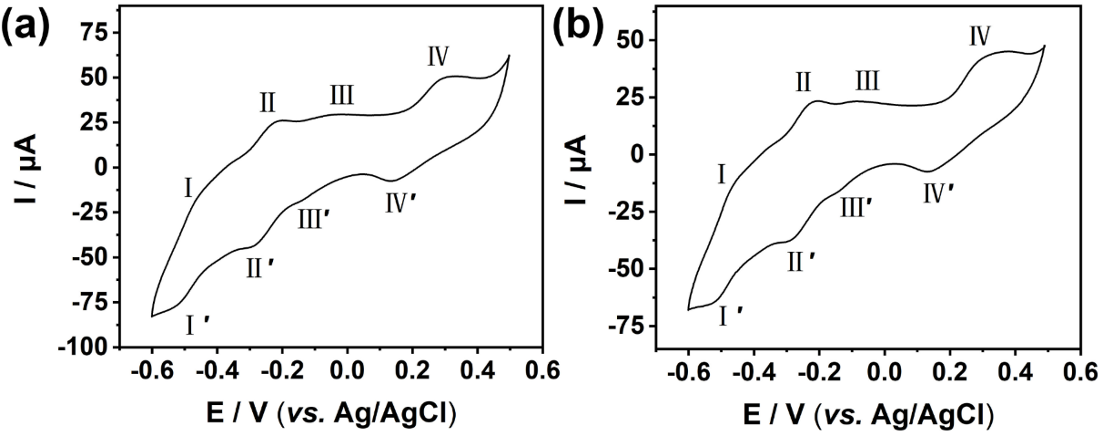


**Supplementary Figure 9 |** CV curves of (a) **NNU-13** and (b) **NNU-14** on glassy carbon electrode were recorded at 100 mV s^-1^ under the H_2_O/TEOA (14:1 V/V, 30 ml) solution injected CO_2_ (30 min); after injecting CO_2_, the pH of solution is 7.3. Catalyst ink was prepared by mixing 5 mg powders into water (450 μL) containing 5 wt % Nafion (50 μL) and then ultrasonically dispersed. Then, an aqueous dispersion was transferred onto the clean-washed glassy carbon electrode (5 μL) and dried in air at room temperature before electrochemical experiments for 30 min. The electrochemical test was carried out on the electrochemical workstation (Bio−Logic) using the standard three−electrode configuration (Ag/AgCl electrode and Pt wire acted as the reference and counter electrode, respectively).


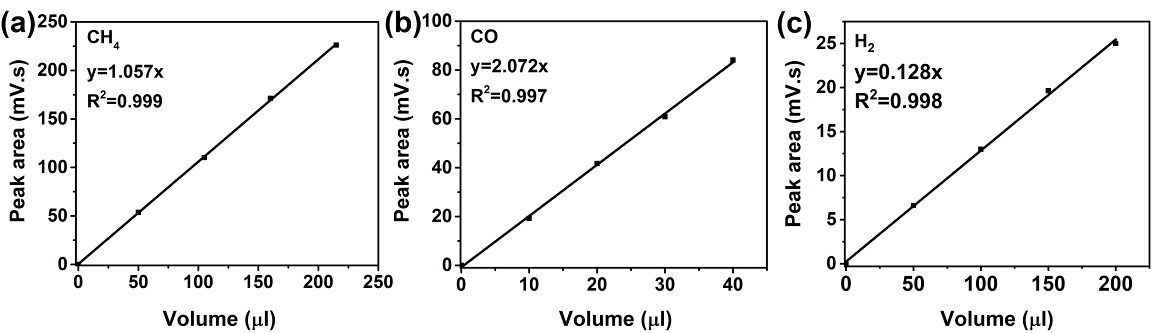


**Supplementary Figure 10** **|** GC calibration lines of (a) CH_4_, (b) CO and (c) H_2_.


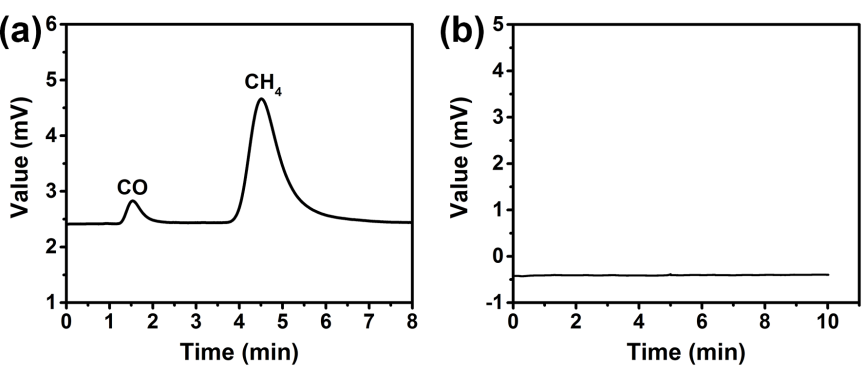


**Supplementary Figure 11** **|** (a) The gas products including CH_4_ and CO were detected by gas chromatogram, (b) and the TCD dectector reveals that there is no H_2_ generation.


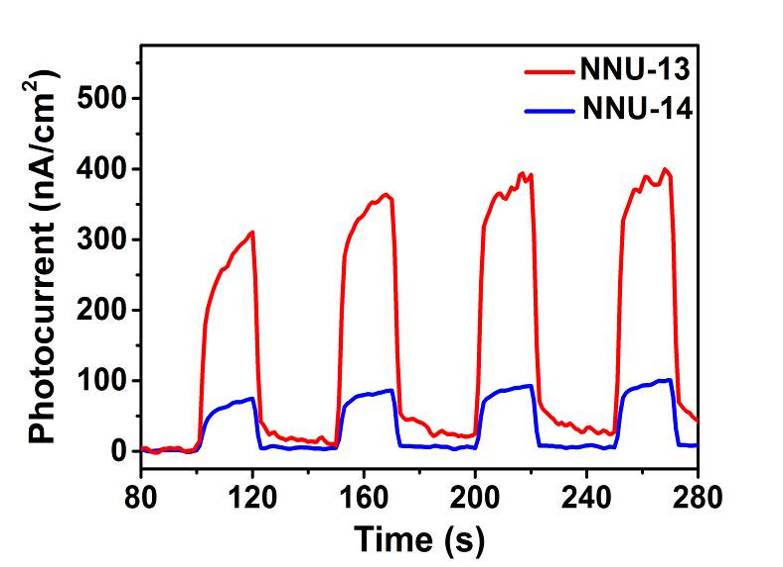


**Supplementary Figure 12** **|** Transient photocurrent vs time profiles of **NNU-13** and **NNU-14**, which were coated on the glassy carbon electrode in a Tris-HCl electrolyte (0.1 M) at a bias of 0.0 V, with and without light irradiation.


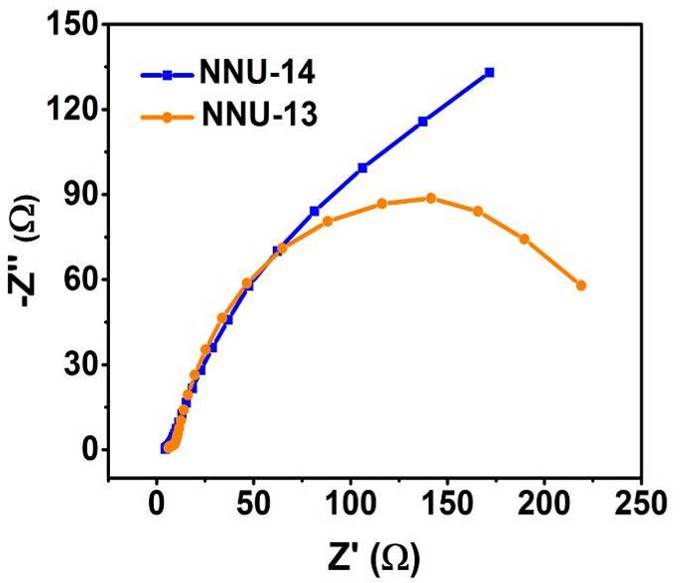


**Supplementary Figure 13** **|** Nyquist plots of catalysts (**NNU-13** and **NNU-14**) over the frequency ranging from 1000 kHz to 0.1 Hz.


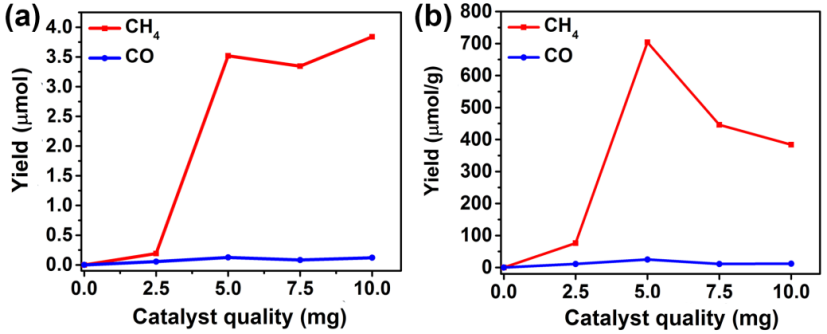


**Supplementary Figure 14** **|** The influence of different qualities for (a) **NNU-13** and (b) **NNU-14** on the activity of photoconversion CO_2_ reaction.


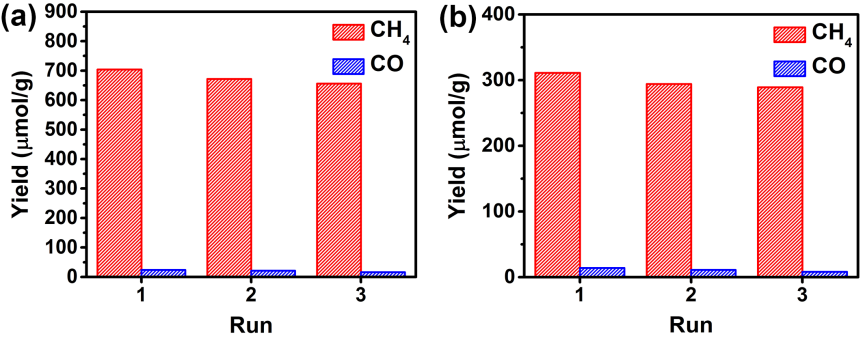


**Supplementary Figure 15** **|** CH_4_ production amounts of repeated photocatalytic CO_2_ reduction reactions by using (a) **NNU-13** and (b) **NNU-14** as catalyst.


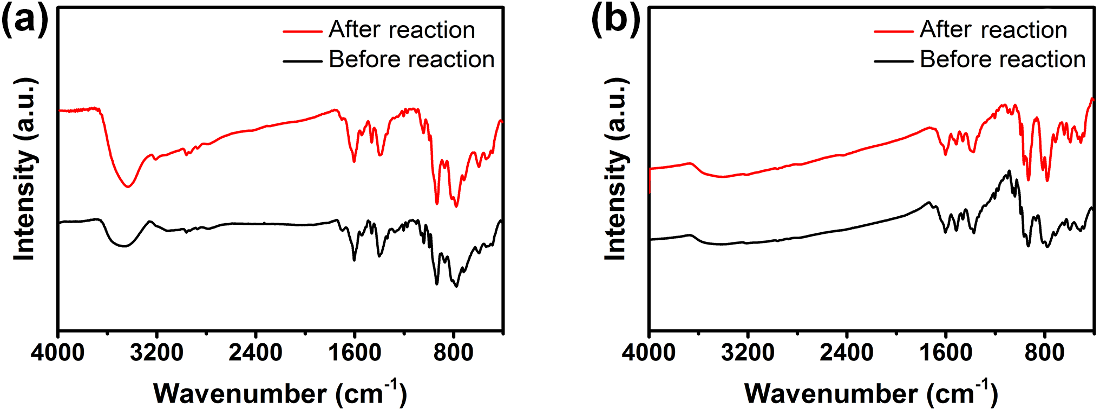


**Supplementary Figure 16** **|** The IR spectra of (a) **NNU-13** and (b) **NNU-14** before and after photocatalytic reaction, respectively. IR ( NNU-13, KBr pellets, ν/cm^−1^): 3461 (m), 3102 (w), 2956 (w), 1706 (w), 1602 (s), 1404 (s), 1340 (m), 1269 (w), 1206(w), 930 (m), 935 (s), 779 (s), 592 (w). IR (NNU-14, KBr pellets, ν/cm^−1^): 3419 (m), 3193 (w), 2955 (w), 1708 (w), 1061 (s), 1522 (s), 1456 (w), 1363 (s), 978 (s), 925 (s), 806 (s), 780 (s), 713 (m), 593 (m), 501 (m). For both of **NNU-13** and **NNU-14**, the peak at about 1700 cm^-1^ is assigned to carboxyl of TCPP. The peaks in the range of 1620-1420 cm^-1^ are assigned to the porphyrin ring skeleton. As we can see, the IR spectra of sample have no noticeable change before and after photocatalytic CO_2_ reduction reaction.


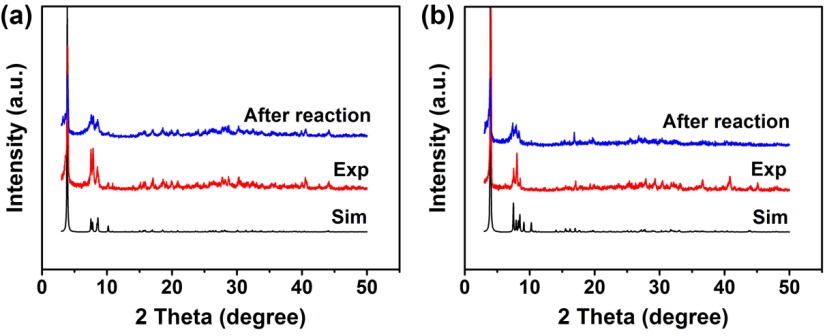


**Supplementary Figure 17** **|** The PXRD patterns of (a) **NNU-13** and (b) **NNU-14** before and after photocatalytic reaction. “Sim” represents the simulated pattern, and “Exp” represents the pattern of as-synthesized sample.

**
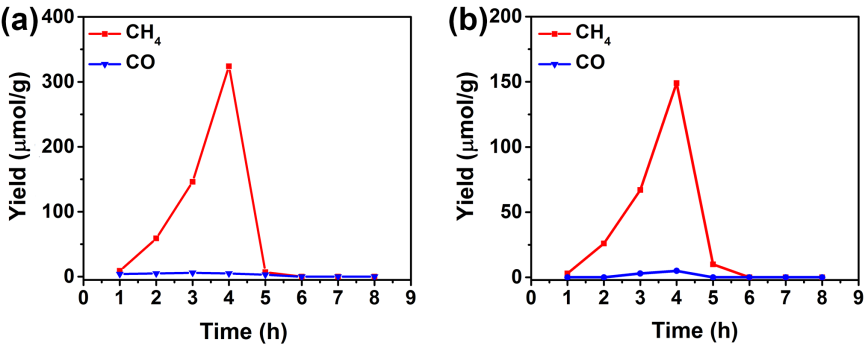
**

**Supplementary Figure 18** **|** The photocatalytic reaction of the filtrate was performed when (a) **NNU-13** or (b) **NNU-14** as the photocatalyst. The gas products were produced as a function of the visible-light irradiation time, and four hours later for **NNU-13** or seven hours later for **NNU-14** the catalyst was removed from the reactor.

**
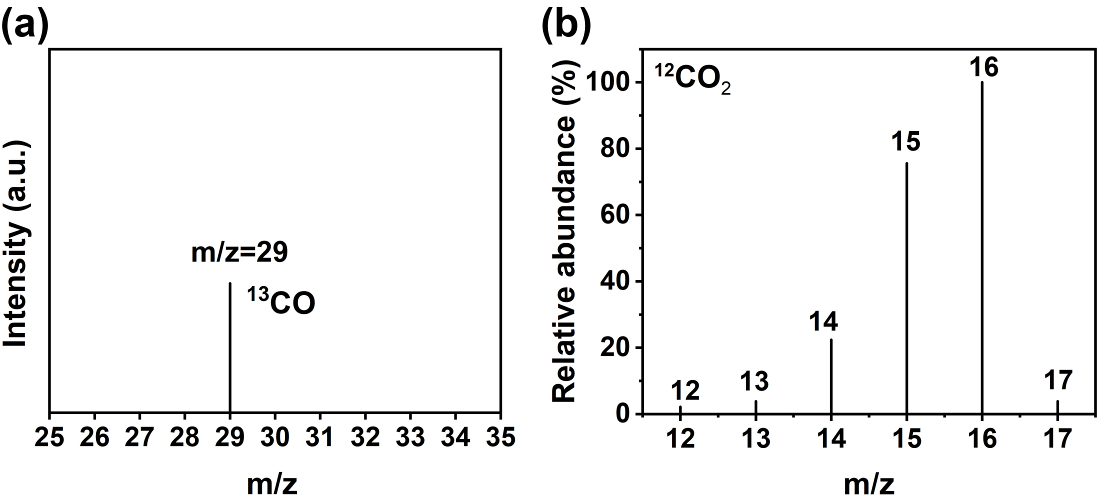
**

**Supplementary Figure 19** **|** (a) the Mass spectra of ^13^CO recorded under a ^13^CO_2_ atmosphere. (b) the Mass spectra of ^12^CH_4_ recorded via single ion monitoring under the normal ^12^CO_2_ atmosphere.


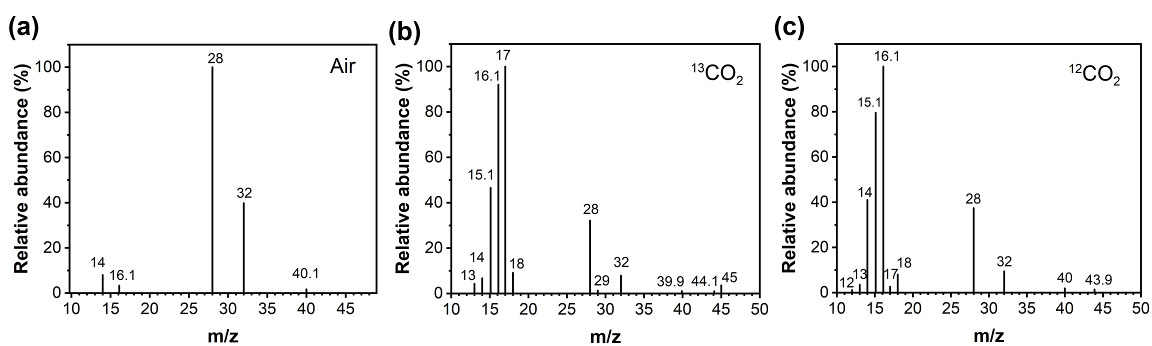


**Supplementary Figure 20** **|** Full scan: (a) the Mass spectra recorded under air atmosphere (all other conditions were the same, but no ^12^CO_2_/^13^CO_2_ was injected.); (b) under a ^13^CO_2_ atmosphere. (c) under a ^12^CO_2_ atmosphere. The photoreduction of CO_2_ reaction occurred in the H_2_O/TEOA (14:1 V/V, 30 ml) solution with photocatalyst (5 mg), and illuminated via a Xe arc lamp with a UV-cutoff filter (420-800 nm). From the GC-MS spectra, under ^12^CO_2_ atmosphere, the peak of 17 with lower abundance comes from HO• in vapor because the water as reaction solvent. Under ^13^CO_2_ atmosphere, the peak of 17 with higher abundance mainly comes from the contribution of ^13^CH_4_. Moreover, when the reaction was carried out under air atmosphere, the peak of 15 would be disappeared. As long as CH_4_ is produced, the appearance of the peak of 15 is accompanied with the production of CH_4_ under ^12^CO_2_ or ^13^CO_2_ atmosphere. As summarized above, the generation of CH_4_ is originated from the CO_2_ photoreduction.


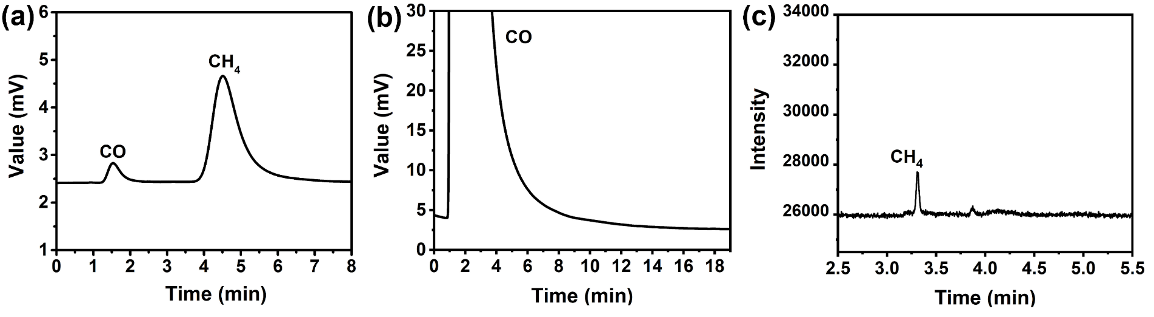


**Supplementary Figure 21** **|** Taking **NNU-13** for example. (a) The gas products including CH_4_ and CO were detected by gas chromatogram under CO_2_ atmosphere. When the experiment was carried out under CO atmosphere instead of CO_2_ atmosphere, (b) CO and (c) CH_4_ were recorded on two gas chromatogram. Because when the CO atmosphere replaced CO_2_ atmosphere as reactant, the peak of large amount of CO would broaden and resulted in covering the peak of the small amount of CH_4_. (a) and (b) were obtained by gas chromatography (GC-7900, CEAULIGHT, China, column type: TDX-01) equipped with a flame ionization detector (FID). (c) was obtained by gas chromatography equipped with FID detector without reborner (Agilent 7820, column type: 19095p-QO4). Therefore, CH_4_ can be detected without interference under CO atmosphere.


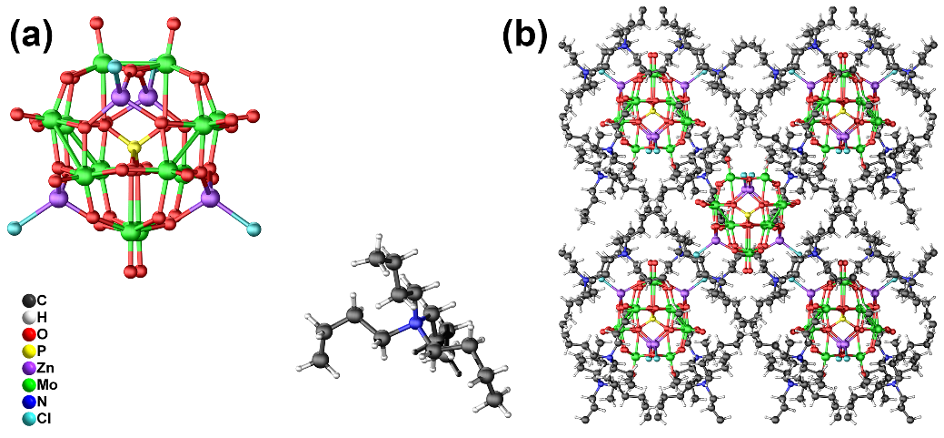


**Supplementary Figure 22 |** (a) Ball-and-stick representation of **NENU-499**. (b) Supramolecular stacking of **NENU-499**.


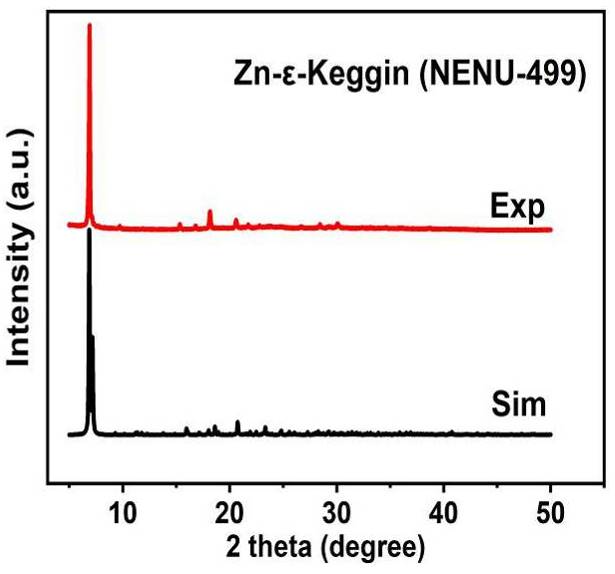


**Supplementary Figure 23** **|** PXRD patterns of Zn-ε-Keggin (**NENU-499**).


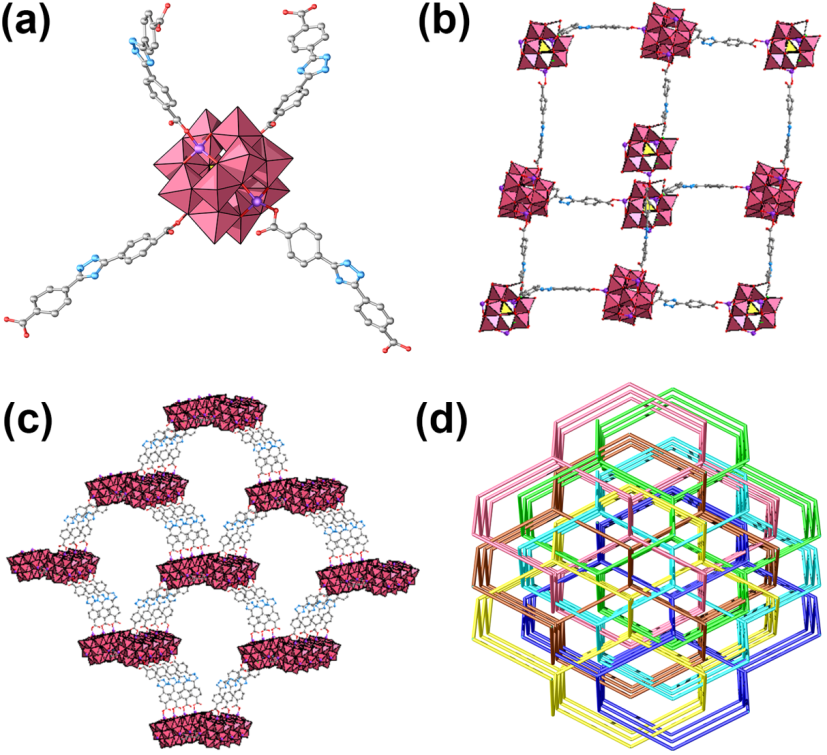


**Supplementary Figure 24** **|** Summary of the structure of **NNU-12**: (a) Zn-ε-Keggin unit and BCPT^2−^ fragment as building blocks, (b) the single diamond illustration, (c) 3D framework, (d) six-fold interpenetrated structure with **dia** topology.


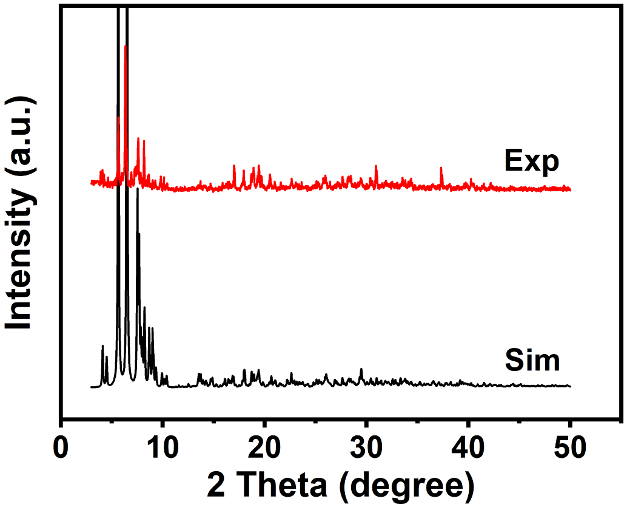


**Supplementary Figure 25** **|** PXRD patterns of **NNU-12**. “Sim” represents the simulated pattern, and “Exp” represents the pattern of as-synthesized sample.


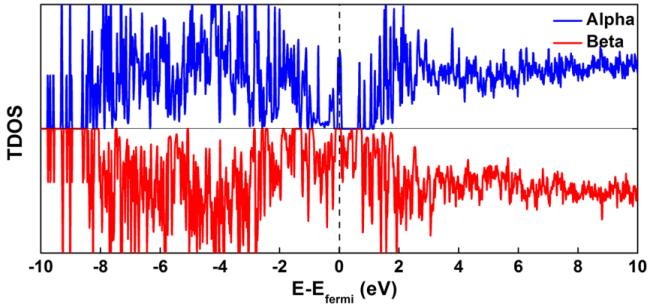


**Supplementary Figure 26** **|** Total density of states (TDOS) of **NNU-13**.


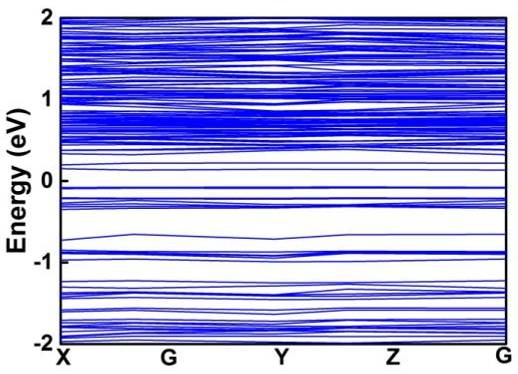


**Supplementary Figure 27** **|** The band structure of **NNU-13**.

**Supplementary Table 1**. Crystal data and structure refinements for **NNU-14**. **NNU-13** have been synthesized in our previous work [8].

| **Compounds** | **NNU-14** |
| --- | --- |
| **Empirical formula** | C_48_H_28_Mo_24_N_4_O_90_P_2_Zn_9_ |
| **Formula weight** | 5053.57 |
| **Crystal system** | Monoclinic |
| **Space group** | *C*2*/m* |
| ***a* (Å)** | 17.394(5) |
| ***b* (Å)** | 23.588(6) |
| **c (Å)** | 23.111(6) |
| ***α* (°)** | 90 |
| ***β* (°)** | 105.038(3) |
| ***γ* (°)** | 90 |
| ***V* (Å^3^)** | 9158(4) |
| ***Z*** | 2 |
| ***D*_calc_(Mg·m^-3^)** | 1.833 |
| **Abs.coeff.(mm^-1^)** | 2.820 |
| ***F*(000)** | 4744.0 |
| **Reflns collected** | 31773 |
| **Independent reflns** | 8538 |
| **GOF** **on *F*^2^** | 1.044 |
| ***R*_int_** | 0.0561 |
| ***R_1_*[*I* > 2*σ*(*I*)]^a^** | 0.0574 |
| ***wR_2_* [*I* > 2*σ*(*I*)]^a^** | 0.1566 |
| ***R*_1_(all data)^b^** | 0.0800 |
| ***wR*_2_(all data)^b^** | 0.1718 |

^a^ *R*_1_ =Σ||*F_o_*| – |*F_c_*||/Σ|*F_o_*|. ^b^ *wR*_2_ = |Σw(|*F_o_*|^2^ – |*F_c_*|^2^)|/Σ|w(*F_o_*^2^)^2^|^1/2^.

CCDC numbers: 1811860 (**NNU-13**) and 1811861 (**NNU-14**)

**Supplementary Table 2a**. The selected bond lengths (Å) for **NNU-14**.

| P1 | O13 | 1.577(8) | Mo5 | O23 | 1.026(5) |
| --- | --- | --- | --- | --- | --- |
| P1 | O14 | 1.564(8) | Mo6 | Mo7 | 2.6181(11) |
| P1 | O15#1 | 1.561(6) | Mo6 | O5 | 2.009(6) |
| P1 | O15 | 1.561(6) | Mo6 | O12 | 1.682(6) |
| Mo1 | Mo2 | 3.1752(17) | Mo6 | O13 | 2.493(6) |
| Mo1 | O3 | 1.669(10) | Mo6 | O19 | 1.951(6) |
| Mo1 | O6#1 | 2.000(6) | Mo6 | O20 | 1.971(6) |
| Mo1 | O6 | 2.000(6) | Mo7 | O10#1 | 2.012(6) |
| Mo1 | O7#1 | 1.829(6) | Mo7 | O15#1 | 2.513(6) |
| Mo1 | O7 | 1.829(6) | Mo7 | O17#1 | 2.012(6) |
| Mo2 | O4 | 1.681(10) | Mo7 | O19 | 1.954(6) |
| Mo2 | O5 | 1.845(6) | Mo7 | O20 | 1.961(6) |
| Mo2 | O5#1 | 1.845(6) | Mo7 | O26 | 1.670(6) |
| Mo2 | O6#1 | 1.995(6) | Zn1 | O1 | 2.029(12) |
| Mo2 | O6 | 1.995(6) | Zn1 | O2 | 2.317(15) |
| Mo3 | Mo4 | 2.6285(11) | Zn1 | O6 | 1.979(6) |
| Mo3 | O7 | 2.017(6) | Zn1 | O8 | 2.003(6) |
| Mo3 | O8 | 1.976(6) | Zn1 | O19#1 | 2.021(6) |
| Mo3 | O9 | 1.677(6) | Zn1 | C12 | 2.565(18) |
| Mo3 | O14 | 2.490(5) | Zn2 | O20#1 | 1.966(6) |
| Mo3 | O16 | 1.972(6) | Zn2 | O20 | 1.966(6) |
| Mo3 | O24#2 | 2.018(4) | Zn2 | O21 | 1.951(8) |
| Mo4 | O8 | 1.962(6) | Zn2 | O22 | 1.966(8) |
| Mo4 | O10 | 2.005(6) | Zn3 | O16 | 1.963(6) |
| Mo4 | O11 | 1.680(6) | Zn3 | O16#1 | 1.964(6) |
| Mo4 | O15 | 2.512(6) | Zn3 | O23 | 1.977(8) |
| Mo4 | O16 | 1.972(6) | Zn3 | O24 | 1.968(8) |
| Mo4 | O18 | 2.021(6) | Zn4 | O1W | 2.669(11) |
| Mo5 | Mo5#1 | 3.2156(16) | Zn4 | N1 | 2.029(14) |
| Mo5 | O17 | 1.807(6) | Zn4 | N1#4 | 2.029(14) |
| Mo5 | O18 | 1.814(6) | Zn4 | N2 | 2.060(13) |
| Mo5 | O22 | 2.021(5) | Zn4 | N2#4 | 2.060(14) |

**Supplementary Table 2b.** The distances between the active Zn center in TCPP pocket and the nearest Zn atom on Zn-ε-Keggin cluster in **NNU-13** and **NNU-14**.

| **NNU-13** | | | **NNU-14** | | |
| --- | --- | --- | --- | --- | --- |
| Zn3 | Zn1#13 | 12.0 | Zn4 | Zn1#7 | 12.9 |
| Zn3 | Zn1#14 | 12.0 | Zn4 | Zn1#8 | 12.9 |
| Zn3 | Zn2#15 | 17.0 | Zn4 | Zn2#9 | 14.2 |
| Zn3 | Zn2#16 | 13.7 | Zn4 | Zn3#9 | 17.9 |
| Zn3 | Zn1#16 | 15.2 | Zn4 | Zn1#11 | 15.1 |
| Zn3 | Zn1#18 | 15.2 | Zn4 | Zn1#12 | 15.1 |
| Zn3 | Zn2#13 | 10.7 | Zn4 | Zn2#10 | 10.9 |
| Zn3 | Zn2#17 | 10.7 | Zn4 | Zn3#10 | 10.1 |
|  |  |  | Zn4 | Zn1#13 | 11.0 |
|  |  |  | Zn4 | Zn1#15 | 11.0 |
|  |  |  | Zn4 | Zn2#13 | 16.0 |
|  |  |  | Zn4 | Zn3#13 | 13.1 |

#: Symmetric operation.

**Supplementary Table 3a.** Conditional exploration for **NNU-13** in photoreduction reaction of CO_2_.

| **Photocatalysts** | **injection gas** | **Illumination** | **TEOA** | **CH_4_ (μmol g^-1^)** | **CO (μmol g^-1^)** | **H_2_ (μmol g^-1^)** |
| --- | --- | --- | --- | --- | --- | --- |
| **NNU-13** | CO_2_ | 6 h | 2 ml | 704 | 25 | not detectable |
| **no** | CO_2_ | 6 h | 2 ml | not detectable | not detectable | not detectable |
| **NNU-13** | N_2_ | 6h | 2 ml | not detectable | not detectable | 11 |
| **NNU-13** | CO_2_ | no | 2 ml | not detectable | not detectable | not detectable |
| **NNU-13** | CO_2_ | 6 h | no | not detectable | not detectable | not detectable |

Reaction conditions: 1. NNU-13 (5 mg), solvent (H_2_O:28ml), TEOA (2 mL), CO_2_ (1 atm), λ > 400 nm, illumination time (6h). 2. Without NNU-13. 3. CO_2_ was replaced by N_2_. 4. In the dark. 5. Without TEOA .

**Supplementary Table 3b.** Conditional exploration for **NNU-14** in photoreduction reaction of CO_2_.

| **Photocatalysts** | **injection gas** | **Illumination** | **TEOA** | **CH_4_ (μmol g^-1^)** | **CO (μmol g^-1^)** | **H_2_ (μmol g^-1^)** |
| --- | --- | --- | --- | --- | --- | --- |
| **NNU-14** | CO_2_ | 7 h | 2 ml | 311 | 12 | not detectable |
| **no** | CO_2_ | 7 h | 2 ml | not detectable | not detectable | not detectable |
| **NNU-14** | N_2_ | 7h | 2 ml | not detectable | not detectable | 7 |
| **NNU-14** | CO_2_ | no | 2 ml | not detectable | not detectable | not detectable |
| **NNU-14** | CO_2_ | 7 h | no | not detectable | not detectable | not detectable |

Reaction conditions: 1. **NNU-14** (5 mg), solvent (H_2_O:28ml), TEOA (2 mL), CO_2_ (1 atm), λ > 420 nm, illumination time (6h). 2. without **NNU-14**. 3. CO_2_ was replaced by N_2_. 4. In the dark. 5. Without TEOA.

**Supplementary Table 4**. Comparisons of title POMCFs with coordination frameworks in CO_2_ photoreduction system.

| **Photocatalysts** | **Light (nm)** | **Reaction time (h)** | **product** | **Yield (μmol g^-1^)** | **References** |
| --- | --- | --- | --- | --- | --- |
| **NNU-13** | **λ > 420** | **6** | **CH_4_**  **CO** | **704**  **25** | **This work** |
| **NNU-14** | **λ > 420** | **7** | **CH_4_**  **CO** | **311**  **12** | **This work** |
| Cu_3_(BTC)_2_@TiO_2_ | λ < 400 | 4 | CH_4_ | 11 | *Adv. Mater.*, **2014**; 26: 4783–4788. |
| TiO_2_-Mg-CPO-27 | λ = 365 | 10 | CH_4_  CO | 40.9  23.5 | *Appl.Catal. B Environ.,* **2016**; 183: 47-52. |
| MOF-525 | λ > 400 | 6 | CH_4_  CO | 37  384 | *Angew. Chem. Int. Ed.*, **2016**; 55: 14310 –14314. |
| MOF-525-Zn | λ > 400 | 6 | CH_4_  CO | 70  670 |
| MOF-525-Co | λ > 400 | 6 | CH_4_  CO | 221  1204 |
| ZrPP-1-Co | λ > 420 | 15 | CH_4_  CO | 8  210 | *Adv. Mater.*, **2017**, 1704388. |
| TiO_2_-Co-ZIF-9 | λ > 200 | 10 | CO  CH_4_  H_2_ | 88  20  26 | *J. Mater. Chem. A* **2016**; 4: 15126. |
| PCN-222 | λ > 420 | 10 | HCOO^–^ | 600 | *J. Am. Chem. Soc.* **2015**; 137: 13440. |
| MIL-125(Ti) | λ< 365 | 10 | HCOO^–^ | 42.8 | *Angew. Chem., Int. Ed.* **2012**; 51: 3364. |
| Pt/NH_2_-MIL-125(Ti) | λ > 420 | 8 | HCOO^–^ | 259.2 | *Chem. Eur. J.* **2014**; 20: 4780. |
| Au/NH2-MIL-125(Ti) | λ > 420 | 8 | HCOO^–^ | 130.4 |
| NH_2_-UiO-66(Zr) | λ > 420 | 10 | HCOO^–^ | 264 | *Chem. Eur. J.* **2013**;19: 14279. |
| CNNS-UiO-66(Zr) | λ > 400 | 6 | CO | 17.4 | *Adv. Funct. Mater.* **2015**; 25: 5360*.* |
| MIL-101(Fe) | λ > 420 | 8 | HCOO^–^ | 1180 | *ACS Catal.* **2014**; 4: 4254. |
| MIL-53(Fe) | λ > 420 | 8 | HCOO^–^ | 594 |
| MIL-88(Fe) | λ > 420 | 8 | HCOO^–^ | 180 |
| {Cd_3_[Ru(5,5′-dcbpy)_3_]_2_ 2(Me_2_NH_2_)}*n* | λ > 420 | 6 | HCOO^–^ | 405 | *Inorg. Chem.* **2015**; 54, 8375. |
| NNU-28 | λ > 420 | 10 | HCOO^–^ | 528 | *J. Mater. Chem. A* **2016**; 4, 2657. |
| g-C_3_N_4_-Co-ZIF-9 | λ > 420 | 2 | CO  H_2_ | 990 157.2 | *Phys. Chem. Chem. Phys.* **2014**; 16, 14656. |
| [Ru(bpy)_3_] Cl_2_-Co-ZIF-9 | λ > 420 | 0.5 | CO  H_2_ | 20.9  15.0 | *, Angew. Chem., Int. Ed.* **2014**; 53, 1034. |
| [Ru(bpy)_3_] Cl_2_-Zn-ZIF-8 | λ > 420 | 0.5 | CO  H_2_ | 1.0  1.2 |
| [Ru(bpy)_3_] Cl_2_-Co-ZIF-67 | λ > 420 | 0.5 | CO  H_2_ | 29600  14800 | *Phys. Chem. Chem. Phys.* **2014**; 16, 14656. |
| [Ru(bpy)_3_]Cl_2_-MOF-253-Ru(5,5′-dcbpy) (CO)_2_Cl_2_ | λ > 420 | 8 | CO  H_2_  HCOO^–^ | 548  382.4 1646.4 | *Chem. Commun.* **2015**; 51, 2645. |
| Al PMOF | λ > 420 | – | CH_3_OH | 37.5 | *ACS Appl. Mater. Interfaces* **2013**; 5, 7654. |
| Al PMOF embedded Cu^2+^ | λ > 420 | – | CH_3_OH | 262.6 |
| Zn_2_GeO_4_-Zn-ZIF-8 | – | 10 | CH_3_OH | 2.2 | *J. Mater. Chem. A* **2013**; 1, 11563. |

**Supplementary Table 5.** ICP-MS characterization for the as-synthesized **NNU-13** and **NNU-14** crystal samples.

| **Photocatalysts** | **Co (wt%)** | **Fe (wt%)** |
| --- | --- | --- |
| **NNU-13** | ＜detection limit | ＜detection limit |
| **NNU-14** | ＜detection limit | ＜detection limit |

The ICP-MS results were measured by Agilent 7700 with detection limit of 1ng/mL.

**Supplementary Table 6.** Some results of the photocatalytic CO_2_ reduction reactions.

| **Photocatalysts** | **TON** | | **TOF (10^-6^) S^-1^** | | **Φ CH_4_ (%)** |
| --- | --- | --- | --- | --- | --- |
|  | **CH_4_** | **CO** | **CH_4_** | **CO** |  |
| **NNU-13** | 3.56 | 0.12 | 164.80 | 5.56 | 0.04 |
| **NNU-14** | 1.57 | 0.06 | 62.30 | 2.38 | 0.02 |

TON = mole of product / mole of catalyst

TOF = TON / reaction time

The apparent quantum yield of CH_4_:

ΦCH_4_ = [8×(number of the produced molecule) / (number of photons)] ×100%

Light intensity (I) = 30 mW/cm^2^. Area (S) = 19.23 cm^2^. λ = 550 nm. Reaction time is 6 hour.

*h* is Planck constant = 6.62606957×10^-34^ J·s , *c* is velocity of light = 3×10^8^ m/s.

NA=6.02214076×10^23^.

photon flux = (I×S) / (*h*×*c* / λ) = 2.64×10^-6^ mol/s.

Note that the apparent quantum yield calculation does not consider the catalyst amount and many other aspects (such as the transparency of the reaction container), meaning that only the experiments carried out with the same setting are suitable for comparison of the apparent quantum yields.

**Supplementary Table 7.** The control experiments for Zn-ε-Keggin, Zn-TCPP, the mixture of Zn-ε-Keggin and Zn-TCPP, and **NNU-13**.

| **Photocatalysts** | **CH_4_ μmol g^-1^** | **CO μmol g^-1^** | **H_2_ μmol g^-1^** |
| --- | --- | --- | --- |
| **Zn-ε-Keggin** | Not detectable | 370 | Not detectable |
| **Zn-TCPP** | Not detectable | 16 | Not detectable |
| **Zn-ε-Keggin and Zn-TCPP** | 40 | 22 | 6.9 |
| **NNU-13** | 704 | 25 | Not detectable |

**Supplementary Table 8.** Gas product species and CH_4_ selectivity was listed using different Photocatalysts for CO_2_ conversion.

| **Photocatalysts** | **CH_4_ μmol g^-1^** | **CO μmol g^-1^** | **Selectivity of CH_4_** |
| --- | --- | --- | --- |
| **NNU-13** | 704 | 25 | 96.6% |
| **NNU-14** | 311 | 12 | 96.2% |
| **NNU-12** | 13 | 3 | 81.3% |
| **MOF-525** | 37 | 384 | 8.8% |
| **MOF-525-Zn** | 70 | 670 | 9.5% |
| **MOF-525-Co** | 221 | 1204 | 15.5% |
| **ZrPP-1-Co** | 7.5 | 210 | 3.4% |

**Supplementary References**

1. Qin J-S, Du D-Y and Guan W *et al*. Ultrastable polymolybdate-based metal–organic frameworks as highly active electrocatalysts for hydrogen generation from water. *J. Am. Chem. Soc.* 2015; **137**: 7169-7177.

2. Feng DW, Gu ZY and Li JR *et al*. Zirconium-metalloporphyrin PCN-222: mesoporous metal-organic frameworks with ultrahigh stability as biomimetic catalysts. *Angew. Chem. Int. Ed.* 2012; **51**: 10307-10310.

3. Clark S-J, Segall M-D and Pickard C-J *et al*. First principles methods using CASTEP. *Z. Kristallogr.* 2005; **220**: 567-570.

4. Hohenberg P and Kohn W. Inhomogeneous electron gas. *Phys. Rev.* 1964; **136**: B864-B871.

5. Kohn W and Sham L-J. Self-consistent equations including exchange and correlation effects. *Phys. Rev.* 1965; **140**: A1133-A1138.

6. Perdew J-P, Burke K and Ernzerhof M. Generalized gradient approximation made simple. *Phys. Rev. Lett*. 1996; **77**: 3865-3868.

7. McNellis E-R, Meyer J and Reuter K. Azobenzene at coinage metal surfaces: role of dispersive van der waals interactions. *Phys.Rev.* 2009; B 80: 205414.

8. Wang Y-R, Huang Q and He C-T *et al.* Oriented electron transmission in polyoxometalate-metalloporphyrin organic framework for highly selective electroreduction of CO_2_. *Nat. Commun.* 2018; **9**: 4466.
